# Associations between perceptions of e-cigarette advertising and interest in product trial amongst US adult smokers and non-smokers: results from an internet-based pilot survey

**DOI:** 10.1186/s12971-015-0039-6

**Published:** 2015-06-12

**Authors:** Danielle M. Smith, Maansi Bansal-Travers, Richard J. O’Connor, Maciej L. Goniewicz, Andrew Hyland

**Affiliations:** Department of Health Behavior, Roswell Park Cancer Institute, Elm & Carlton Streets, Buffalo, NY 14263 USA

**Keywords:** Electronic cigarettes, Advertising, Tobacco, Marketing, Smoking

## Abstract

**Background:**

Electronic cigarettes (e-cigarettes) have risen in popularity in the U.S. While recent studies have described the prevalence and demographics of e-cigarette users, few studies have evaluated the impact of advertising on perceptions and interest in trial. This pilot study was conducted to assess whether exposure to ads for e-cigarettes or a comparison product (snus), elicited differences in interest to try e-cigarettes between smokers and non-smokers.

**Methods:**

A web-based survey was completed by 600 respondents, aged 18–65, recruited from an internet panel in the U.S. Respondents answered questions assessing tobacco use, and then viewed nine magazine ads for Blu e-cigarettes or Camel snus, a low-nitrosamine smokeless tobacco product, in random order. After viewing each ad, respondents were asked a series of questions about their perceptions, beliefs, attitudes, and interest in trial. At the end, respondents were asked to choose a free sample product from the following options: an e-cigarette, smokeless tobacco (SLT), pack of cigarettes, or no product.

**Results:**

Ad receptivity scores did not appear to be influenced by ad theme; differences existed between smokers and non-smokers. Participants exposed to e-cigarette ads more frequently reported favorable product attitudes compared to participants exposed to snus ads. Cigarette smokers in the e-cigarette condition were more likely to report interest in trying e-cigarettes compared to non-smokers in that condition (*p*-value < 0.001). Six percent of non-smokers exposed to e-cigarette ads reported interest in trying e-cigarettes. E-cigarettes were the most popular product selected to sample (34 %), followed by cigarettes (8 %) and SLT (3 %); 331 respondents (55 %) chose no product. Participants randomized to the e-cigarette ad group were significantly more likely to choose an e-cigarette at product selection (*p*-value = 0.014). Within the e-cigarette condition, 71 % of smokers selected an e-cigarette at product selection, compared to 25 % of non-smokers; smoking status was significantly associated with sample product selection (*p*-value <0.001).

**Conclusions:**

These findings suggest that exposure to e-cigarette ads may be associated with interest in e-cigarette trial, particularly among smokers. Continued exposure to advertising in magazines, on television, and at the point-of-sale may have an impact on willingness to receive promotional products or intention to try e-cigarettes.

**Electronic supplementary material:**

The online version of this article (doi:10.1186/s12971-015-0039-6) contains supplementary material, which is available to authorized users.

## Background

E-cigarettes are a novel phenomenon. These devices provide nicotine to users in the form of an aerosol vapor, which is promoted as a reduced risk form of delivery. Awareness of e-cigarettes and other electronic nicotine delivery devices is increasing. Recent nationally representative studies conducted among the U.S. adult general population have shown that awareness of e-cigarettes has risen from approximately 40 % in 2010, to nearly 60 % in 2011 [1, 2]. A survey conducted from 2010–2011 among current and former U.S. cigarette smokers reported that 73 % of responders were aware of e-cigarettes, denoting a heighted awareness of these products among this population subgroup [3].

The increased awareness of e-cigarettes may be linked to a corresponding increase in media presence; including ads in magazines, television, and strategic product placement of e-cigarettes in Hollywood productions. For example, e-cigarettes have been used by celebrities in commercials, print ads, movies, and television shows [4]. Availability of e-cigarettes is also increasing in a variety of retail outlets, including gas stations, convenience stores, mall kiosks, vapor lounges, and over the Internet [5–7]. In addition to independent e-cigarette manufacturers, the tobacco industry has also expanded their product line to include e-cigarettes (e.g., RJ Reynolds’ production of Vuse, Altria’s production of MarkTen). Using strategies similar to those they have successfully employed in the past to market and distribute conventional cigarettes, Lorillard was the first to market Blu e-cigarettes on TV [8]. These ads showed celebrities using e-cigarettes in places where smoking is banned by clean indoor air legislation. Manufacturer investments in advertising and marketing for e-cigarettes are increasing. Recent reports have found that advertising and marketing of e-cigarettes by e-cigarette manufacturers across all media channels exceeded $82 million in 2013 and continues to increase [9–11]. With increased awareness resulting from increased exposure, Lorillard expanded the distribution of Blu electronic cigarettes to over 50,000 retailers by the fourth quarter of 2012, resulting in net sales for this time period totaling approximately $39 million [12].

While e-cigarettes may be beneficial for individual smokers as a harm reduction device, it is yet unclear if these devices yield a positive or negative public health impact. The use of e-cigarettes as a complete replacement for conventional cigarettes or as a quit aid could result in a net public health gain [13, 14]. However, use of e-cigarettes in conjunction with cigarettes or other tobacco products, or introduction of nicotine to non-smokers would have an overall negative impact on public health. For example, a recent study conducted among a sample of U.S. adults demonstrated that current daily and non-daily smokers are more likely to concurrently use e-cigarettes than those who had never smoked cigarettes, suggesting potential for dual-use in lieu of complete substitution. The authors of this study also found that nearly one-third of current e-cigarette users are non-smokers, including both former and never users of conventional cigarettes [15].

Evidence from multiple U.S. Surgeon General’s reports support the notion that tobacco advertising and promotion can influence risk perception, trial, and use of tobacco products in both adults and youth [16–18]. Such evidence contributed to the establishment of regulations to limit tobacco advertising and marketing in the United States [19–21]. Current restrictions on tobacco advertising in the U.S. include a bans on outdoor marketing (including billboards and public transit), advertising that targets youth (including use of cartoons), sponsorship of sporting or other public events, and ads on television and radio. Although e-cigarettes are planned for regulation as a ‘tobacco product’ by the Center for Tobacco Products in the U.S. Food and Drug Administration (FDA), currently, e-cigarette products are not subject to the same promotional restrictions as cigarettes. (For example, e-cigarettes advertisements are currently aired on television.) [22] As of November 2014, at least 40 state level authorities have banned the sale of e-cigarettes to minors, and over 200 jurisdictions have amended their clean indoor air legislation to include the prohibition of e-cigarettes in places where traditional cigarette smoking is not allowed. [23–25] Forthcoming restrictions on e-cigarettes in the U.S. could include restrictions on advertising [22].

While an increasing number of recently published studies have described awareness and use of e-cigarettes [1–3], very few studies have evaluated associations between advertising and e-cigarette perceptions and interest in trial. Such studies have largely focused among the impact of advertising and trial among smokers [26], but we are not aware of studies that have expanded this concept to include non-smokers. With the increasing number of tobacco companies expanding their product lines to include e-cigarettes, as well as the increasing number of independent e-cigarette manufacturers in the market, there is concern that increased advertising, coupled with the sharp increase in availability, may influence current tobacco users and non-users (including never and former users) to consider trial and adoption of e-cigarettes.

This pilot study aimed to explore issues related to e-cigarette advertising and the potential impact such advertisements may have on the public’s willingness to try e-cigarettes among current smokers and non-smokers. Namely, we sought to assess whether exposure to ads for e-cigarettes or a comparison product (snus), showed differences in interest to try e-cigarettes among both cigarette smokers and non-smokers.

## Methods

### Study procedures

**Fig. 1:**
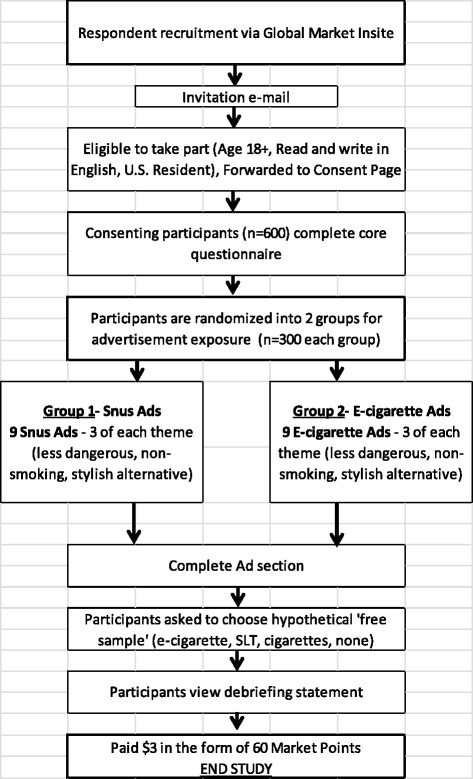
Study design

This web-based survey was conducted in March 2013 among cigarette smokers and non-smokers, including current users of e-cigarettes (see schema, Fig. 1). Sample members were recruited through a web panel managed by Global Market Insite, an organization specializing in the administration of online surveys (http://www.lightspeedgmi.com/). Eligible participants answered a series of preliminary questions to confirm that they were between ages 18 and 65, current United States residents, and able to read and write in English. After determining eligibility, participants were directed to an informed consent screen, clicking a button indicating consent. Respondents completed a core series of questions adapted from a similar study assessing tobacco use, past quit attempts, use of nicotine replacement therapies, and knowledge, attitudes, and beliefs about nicotine-containing products [27]. Next, respondents were randomized to one of two exposure groups to view magazine ads for (Blu) e-cigarettes or (Camel) snus. Advertisements belonged to one of three *a priori* themes discerned by raters prior to survey initiation (as described below). Once a participant was randomized to a condition, they were shown nine ads for that product. Within-product presentation sequence of ads was randomized to minimize ordering effects. Participants were asked a series of questions after viewing each ad, as well as a shortened list of product attitudes used in prior research (Table 1) [28–30]. After viewing all ads in their assigned group, participants were asked, “If we had the opportunity to send you a free sample of one of the products listed below, which of the products would you choose?” (response options: an e-cigarette, a tin of smokeless tobacco, a pack of cigarettes, or ‘please do not send me any of these’). At this point, participants were not explicitly made aware by the research team that they would not receive a free sample product. Following the selection of a product, participants were debriefed and informed that ethical guidelines do not allow the researchers to mail free samples of products. The research protocol was approved by the Institutional Review Board at Roswell Park Cancer Institute in Buffalo, NY.

**Table 1:** Ad measures

Perceptions about Ads	Product attitudes
*This ad…*	*Based on what I just saw, I think this product…*
…was clear	…is sophisticated
…had a message that was important to me	…is fun
…made me stop and think	…is satisfying
…made me curious to know if that the ad says is true	…is stupid
…is one I would talk to other people about	…is hard to quit using
…told me something new	…makes me nauseated
…talked down to me	…is for kids
…said things that were hard to believe	…is for adults
	…is something I want to try

### Product advertisements

For this study, Camel snus ads were selected as comparison ads because prior research has indicated that message themes and presentation in print advertising for snus are similar to those for e-cigarettes [31, 32]. Three trained coders who were unaffiliated with the study independently coded each ad on a series of thematic characteristics (Fleiss’s Kappa = 0.55; p_a_ [proportion of agreements to total ratings] = 0.71). Selected themes on which the ads were rated include: 1) less dangerous to health than cigarettes; 2) product use in non-smoking situations; and 3) stylish alternative to tobacco cigarettes. All advertisements were found on the website, “Trinkets & Trash: Artifacts of the Tobacco Epidemic” (www.trinketsandtrash.org) [33]. Advertisements shown to participants can be seen in Additional file 1.

### Measures

#### Product use status

Variables indicating product use were computed for all tobacco products asked about within the questionnaire (cigarettes, smokeless tobacco or SLT, and e-cigarettes). “Current users” for a tobacco product were defined as those individuals who responded “Yes” to the question, “Have you ever used [tobacco product- cigarettes, SLT, electronic cigarettes], even once?” and currently reported using the tobacco product every day or some days. “Ever users” for a tobacco product were defined as those individuals who responded “Yes” to the question, “Have you ever used [tobacco product], even once?”, and currently reported not using the product at all. “Never users” for a tobacco product were defined as those individuals who responded “No” to the question, “Have you ever used [tobacco product], even once?” In analyses examining differences between current cigarette smokers versus current non-smokers, “ever” and “never” smokers were combined into one category.

### Ad receptivity

For each individual ad, we asked the series of statements shown in Table 1 (response options: strongly agree, agree, neither agree nor disagree, disagree, strongly disagree). To indicate favorable perceptions toward each ad, we created a summary score from selected measures: (This ad…was important to me, made me stop and think, made me curious to know if what the ad says is true, is one I would talk to other people about, told me something new; Cronbach’s alpha = 0.98). These measures were chosen as a result of the high internal consistency for responses to the items. For each measure, responses were scored from 0 (strongly disagree) to 5 (strongly agree), then summed together. The scores for each measure were then added to create the summary score for the individual ad. The summary scores for individual ads were then grouped by each of the three ad themes (less dangerous to health than cigarettes; circumvent clean indoor air restrictions; stylish alternative to tobacco cigarettes) and totaled to create an “ad receptivity score” for that set of ads (range: 0–75). We also created a combined ad receptivity score to examine differences for the entire condition, regardless of ad theme (range: 0–225).

### Main outcome measures

We employed two different measures to assess interest in trying e-cigarettes within our sample. The first measure, “intention to try”, was based on the statement: “Based on what I just saw, I think this product is something I want to try.” (response options: strongly agree, agree, neither agree nor disagree, disagree, strongly disagree). To keep our analysis succinct, we opted to recode this Likert measure into a binary variable. The answer options “strongly agree” and “agree” were combined to indicate “intention to try”, while the remaining options were combined to indicate “does not intend to try”.

The second measure, labeled “willingness to receive a free product”, was derived from the question administered for product selection by respondents, which stated, “If we had the opportunity to send you a free sample of one of the products listed below, which of the products would you choose?” (response options: an e-cigarette, a tin of smokeless tobacco, a pack of cigarettes, or ‘please do not send me any of these’).

### Statistical analysis

Descriptive analyses were performed on these pilot data. Pearson chi-square tests were conducted to assess associations between demographic variables, smoking status and main outcome measures (all categorical). Demographic measures included age group (18–24, 25–34, 35–44, 45–54, 55–65), gender (male vs. female), level of education (high school graduate or less, some college, and bachelor’s degree or higher), and racial self-identification (white non-Hispanic, black non-Hispanic, Hispanic, other race non-Hispanic). Independent samples t-tests (equal variance assumed) were used in comparing mean ad receptivity scores, grouped according to cigarette smoking status (current smoker versus current non-smoker) Cramer’s V is reported for effect sizes related to Pearson chi-square tests, while Cohen’s *d* is reported for effect sizes related to independent samples t-tests. Results are reported for four groups of interest: Smokers who viewed ads for e-cigarettes, non-smokers who viewed ads for e-cigarettes, smokers who viewed ads for snus, and non-smokers who viewed ads for snus.

## Results

A total of 46,561 e-mail invitations were sent to GMI panelists, inviting them to take part in the survey. 875 individuals began the survey, of which 600 completed the survey in full (Table 2). Most participants were aged 55–65 (23 %), had some college education (47 %), and identified as being white, non-Hispanic (80 %); the sample had similar proportions of males and females. Relative to national estimates, there were a high proportion of current smokers (31 %), and current SLT users (8 %) in our sample [34]. Among cigarette smokers, prevalence of current e-cigarette use was 21 %, while among current non-smokers only 1 % used e-cigarettes (*χ*^2^ = 144.692, *p*-value < 0.001; Cramer’s V = .491). Current smokeless tobacco use among current cigarette smokers was 17 %, while 4 % of current non-smokers reported use of smokeless tobacco (*χ*^2^ = 32.338, *p*-value < 0.001, Cramer’s V = .232). Ten percent of the total sample reported using more than one tobacco product, while nearly 20 % of our sample consisted of individuals who have at least tried an e-cigarette one time (12 % ever users, 7 % current users).

**Table 2:** Participant demographic characteristics stratified by advertisement viewing condition^a^ (n = 600)

		SNUS advertisements (n = 300)		E-cigarette advertisements (n = 300)			Total sample
		n	%	n	%	*p*-value	%
Gender	Male	144	48	152	51	0.514	49
	Female	156	52	148	49		51
Age	18-24	61	20	45	15	0.405	18
	25-34	49	16	60	20		18
	35-44	61	20	57	19		20
	45-54	61	20	67	22		21
	55-65	68	23	71	24		23
Education	HS Grad/GED or less	55	18	55	18	0.852	18
	Some college	139	46	144	48		47
	Bachelors +	106	35	99	33		34
Race	White	231	77	244	82	0.405	80
	Black	22	7	19	6		7
	Hispanic	29	10	19	6		8
	Other	18	6	15	5		5
Cigarette use	Never Smoker	96	32	77	26	0.168	29
	Ever Smoker	112	37	131	44		41
	Current Smoker	92	31	92	31		31
SLT use	Never User	233	78	232	77	0.909	78
	Ever User	43	14	46	15		15
	Current User	24	8	22	7		8
E-cigarette use	Never User	172	57	165	55	0.7	56
	Ever User	38	13	36	12		12
	Current User	17	6	24	8		7
	Unaware of EC	73	24	75	25		25
Dual/Polyuse	No Tobacco	201	67	198	66	0.367	67
	Single Product	74	25	72	24		24
	Cigs and SLT	9	3	7	2		3
	Cigs and E-cigs	7	2	16	5		4
	SLT and E-cigs	0	0	1	0		0
	Polyuser (Cigs + SLT + EC)	9	3	6	2		3

*P*-value denotes significance resulting from chi-square test of independence between advertisement exposure groups

^a^The percentages presented for the total sample may not equal 100 % due to rounding

### Product attitudes

Participants were asked to respond to a set of questions assessing attitudes about the product shown in their exposure condition (e-cigarette or snus) displayed in Table 1. In general, participants in our study who were exposed to e-cigarette ads rated their product more favorably across this set of measures when compared to participants who were shown ads for snus (Table 3). As one between-condition example, 36 % of participants who viewed e-cigarette ads agreed that the product was “sophisticated”, compared to 14 % of participants who reported the same for snus; we observed a positive, moderately strong relationship between product attitudes and ad exposure grouping. (*χ*^2^ = 46.237, *p*-value < 0.001, Cramer’s V = .278). Table 4 outlines within-condition comparisons of product attitudes according to participant smoking status. Among participants that viewed ads for e-cigarettes, smokers more frequently reported favorable attitudes toward e-cigarettes compared to non-smokers. As one within-condition example for e-cigarettes, 57 % of current smokers reported that e-cigarettes were “sophisticated” compared to 27 % of non-smokers who reported the same. We observed a positive, strong relationship between product attitudes and smoking status (*χ*^2^ = 31.117, *p*-value < 0.001, Cramer’s V = .322). Similar trends were observed among smokers and non-smokers within the snus comparison condition. Among all non-smokers in either condition, more favorable perceptions were observed for e-cigarettes compared to snus.

**Table 3:** Between-condition comparison of product attitudes (n = 600)

	Snus condition % (n = 300)	E-cigarette condition % (n = 300)	*χ*2	*p*-value	Cramer’s *V*
…is sophisticated	14	36	46.24	<.001	0.278
…is fun	15	29	23.73	<.001	0.199
…is satisfying	20	38	34.59	<.001	0.240
…is stupid	48	36	30.12	<.001	0.224
…is hard to quit using	53	31	48.15	<.001	0.284
…makes me nauseated	39	19	49.91	<.001	0.288
…is for kids	8	3	6.06	0.417	0.100
…is for adults	51	73	39.17	<.001	0.256

**Table 4:** Within-condition comparison of product attitudes according to smoking status (n = 600)

	Snus					E-cigarettes				
	Smokers % (n = 92)	Non-smokers % (n = 208)	*χ*2	*p*-value	Cramer’s *V*	Smokers % (n = 92)	Non-smokers % (n = 208)	*χ*2	*p*-value	Cramer’s *V*
…is sophisticated	22	11	23.79	<.001	0.282	57	27	31.12	<.001	0.322
…is fun	23	12	32.24	<.001	0.328	50	20	41.36	<.001	0.371
…is satisfying	35	13	33.00	<.001	0.332	63	27	46.49	<.001	0.394
…is stupid	31	56	29.14	<.001	0.312	19	43	38.96	<.001	0.360
…is hard to quit using	43	57	19.24	0.004	0.253	22	35	17.07	0.004	0.239
…makes me nauseated	29	43	10.39	0.109	0.186	4	26	37.46	<.001	0.353
…is for kids	11	6	6.21	0.4	0.144	7	3	10.56	0.103	0.188
…is for adults	60	47	20.51	0.002	0.261	88	67	22.10	0.001	0.271

### Ad receptivity

**Fig. 2:**
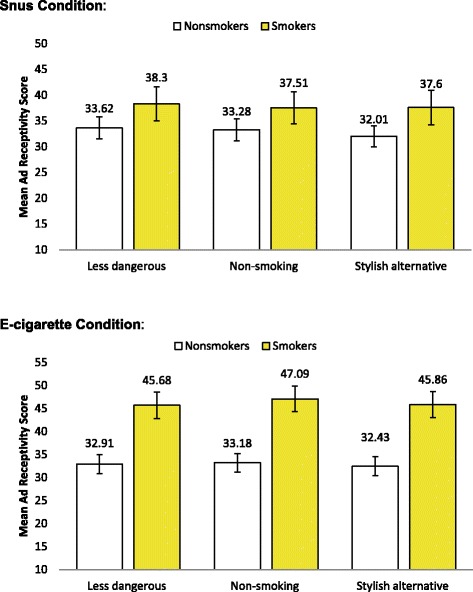
Ad receptivity scores across themes, stratified by ad exposure and smoking status (n = 600). Significant differences in mean ad receptivity scores were observed between current smokers and non-smokers within each ad condition (Independent samples t-test, p-value<0.05). Error bars denote 95 % confidence interval for estimate

Figure 2 displays ad receptivity scores stratified by exposure condition and smoking status. Within the e-cigarette condition, the combined mean ad receptivity score for smokers was 138.63 (SD = 39.09). By contrast, the combined mean ad receptivity score for non-smokers who viewed e-cigarette ads was 98.52 (SD = 44.01); we observed a positive, strong relationship between e-cigarette ad receptivity and smoking status (t(298) = 7.525, *p*-value < 0.001, Cohen’s *d* = 0.872). In the snus comparison condition, the combined mean ad receptivity score for smokers was 113.41 (SD = 45.10), compared to 98.92 (SD = 45.25) for non-smokers. We observed a positive, weak relationship between snus ad receptivity and smoking status (t(298) = 2.561, *p*-value = .011, Cohen’s *d* = 0.297). Mean ad receptivity scores among smokers in the snus condition ranged from 37.51-38.30, while mean ad receptivity scores among smokers exposed to e-cigarette ads ranged from 45.68-47.09.

### Exposure to advertisements and intention to try

**Fig. 3:**
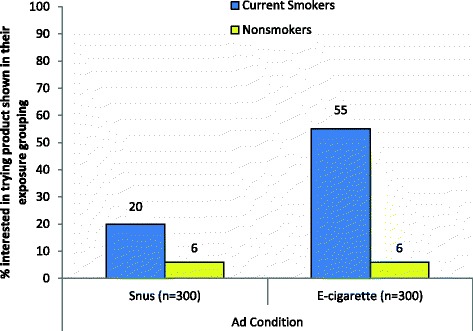
Percentage of respondents reporting intention to try product shown in exposure condition (n = 600). Significant differences in responses were observed between current smokers and non-smokers within each ad condition (Pearson chi-square test, p-value<0.05)

In the combined sample, 94 respondents (16 %) reported that the product shown in their exposure grouping (snus or e-cigarettes) was “something I want to try” (Fig. 3) Among all participants who were shown ads for e-cigarettes, 21 % expressed interest in trying e-cigarettes. Cigarette smokers in the e-cigarette condition were more likely to report interest in trying e-cigarettes compared to non-smokers in that condition (*χ*^2^ = 91.95, *p*-value < 0.001, Cramer’s V = .554). Among all participants in the snus comparison condition, 10 % reported that they intended to try snus and a weaker, yet statistically significant association was observed between smoking status and intention to use snus (*χ*^2^ = 13.49, *p*-value < 0.001, Cramer’s V = .212).

### Exposure to advertisements and willingness to receive a free sample product

**Fig. 4:**
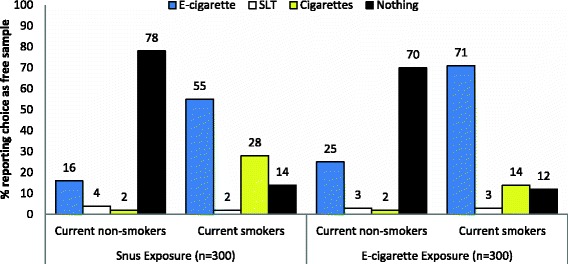
Associations between smoking status and willingness to receive a product, stratified by exposure condition (n = 600). Product selection within each exposure group was significantly associated with smoking status, according to chi-square test of independence (p<0.001)

In the product selection task, over half of the sample (55 %) elected to not be sent a free sample product. E-cigarettes were the most frequently selected tobacco product among all participants, with 34 % reporting this choice. Selection of smokeless tobacco and cigarettes among sample members was low (3 % and 8 %, respectively). Participants exposed to e-cigarettes ads were more likely to select an e-cigarette as their hypothetical free product (39 %) compared to participants who saw ads for snus (28 %). In comparing ad exposure conditions, we observed a statistically significant association between ad exposure grouping and product choice (*χ*^2^ = 10.59, *p*-value = 0.014, Cramer’s V = .133). Within the e-cigarette condition, smokers more frequently selected an e-cigarette at product selection, compared to non-smokers (Fig. 4). We observed a strong statistically significant association between smoking status and product choice within this exposure grouping (*χ*^2^ = 91.78, *p*-value < 0.001, Cramer’s V = .553). Within the snus condition, e-cigarettes remained the most frequently selected tobacco product among responders, with smokers more frequently reporting this choice than non-smokers. Product choice in the snus condition was significantly associated with smoking status (*χ*^2^ = 121.92, *p*-value < 0.001, Cramer’s V = .637).

### Comparing intention to try and willingness to receive a free sample product

In total, 16 % of respondents reported that they intended to try the product shown in their exposure condition. Forty-five percent of respondents elected to receive a sample product (e-cigarette, smokeless tobacco, or a pack of cigarettes). Among smokers in the e-cigarette condition who reported intending to try an e-cigarette, 90 % selected the e-cigarette as their sample product, while 10 % selected another option. Among smokers in the e-cigarette condition who did not report intending to try an e-cigarette, 46 % selected the e-cigarette as their sample product, while 53 % selected another option (*χ*^2^ = 25.53, *p*-value < 0.001, Cramer’s V = .527). One-quarter of non-smokers in the e-cigarette condition selected the e-cigarette as their sample product; 3 % of which previously reported intention to try the product after being shown ads for e-cigarettes. These findings among non-smokers in the e-cigarette condition were not statistically significant. In the snus comparison condition, we did not observe any significant associations between product selection and intention to try e-cigarettes among smokers. Among non-smokers in the snus condition, 5 % of non-smokers previously reported intention to try snus, while 4 % selected smokeless tobacco during product selection. Nearly 16 % of non-smokers in the snus condition chose an e-cigarette during product selection. We observed a statistically significant association between product choice and intention to try snus among non-smokers in the snus condition (*χ*^2^ = 9.25, *p*-value = .026, Cramer’s V = .211).

## Discussion

This pilot study is among the first to test associations between intention to try e-cigarettes and exposure to e-cigarette advertising in a sample of both smokers and non-smokers. The findings from this pilot study suggest that exposure to advertising for e-cigarettes may enhance interest in e-cigarette trial, particularly among cigarette smokers.

In this study, participants who were exposed to e-cigarette advertising were twice as likely to have reported an intention to use e-cigarettes in the future, consistently rated their product more favorably than those exposed to snus ads, and more frequently selected e-cigarettes as their product of choice when offered a free sample product. This could be a result of e-cigarette ad exposure in general, as ads provide a mechanism to communicate awareness of this product. Yet, in our product selection task, participants exposed to snus also more frequently selected e-cigarettes as their chosen sample product, albeit not as frequently as those exposed to e-cigarette ads. We conclude that this could simply indicate a general preference toward e-cigarettes relative to snus, given that e-cigarettes are a relatively novel product. There may be aspects of e-cigarettes (in terms of construction, utility, or other factors) that may enhance appeal as a product that may reduce harm to health. Such aspects are not currently addressed by the data presented in this report, and should be studied in greater detail.

These data suggest that more favorable perceptions toward e-cigarettes existed among all participants, but particularly among current cigarette smokers, who rated e-cigarette ads, interest in trial, and attitudinal measures on e-cigarettes more favorably when compared to snus, a similarly marketed harm reduction product. Smokers exposed to either form of advertising overwhelmingly chose the e-cigarette in the product selection task, yet this was much more frequently observed among smokers in the e-cigarette condition. This product selection occurred over other options, one of which was a pack of cigarettes. Such findings may suggest that e-cigarettes, often proposed as a product that may reduce harm to health, appear to be more favorable to smokers than snus, another frequently proposed harm reduction product. These pilot data do not measure specifically why this may be the case. Snus is a smokeless tobacco product, possessing a different mode of administration for nicotine than e-cigarettes, and smokeless products currently carry health warning labels as mandated by law. Our findings also support the notion of snus posing relatively low appeal to smokers for harm reduction, as demonstrated through previous studies [27, 35]. Factors that may attract smokers to certain tobacco products should be examined more closely through future research.

While we observed more favorable ad receptivity scores for e-cigarette ads compared to snus among smokers, ad receptivity for either tobacco product was relatively similar among non-smokers in both conditions. Additionally, non-smokers in our study generally did not report intention to use either of the products they were exposed to, with 6 % of non-smokers in each group reporting intention to try the product after viewing ads. In the product selection task, non-smokers were more likely to reject the offer of a free sample tobacco product, compared to smokers. Interestingly, a small proportion of non-smokers who did not previously report intention to try e-cigarettes prior to the offer of a free e-cigarette opted to select a free sample e-cigarette. The broader implications of these findings among non-smokers are unclear. With the limited offering of products during product selection, coupled with the absence of measures to indicate reasons for selection of a particular product or participant follow-up over time, we cannot be sure whether non-smokers in our sample chose a free tobacco product for personal use or for some other reason. Future studies should examine such reasons in greater detail.

Our data did not allude to particular advertisement themes (e.g., using e-cigarettes in non-smoking situations) having a strong association with either use intention to use or willingness to receive a free product. It could be that other elements of advertisements not addressed in this study could play a determining factor in influencing interest in these products, a few of which have been demonstrated in other studies. For example, Pepper et al. [26] conducted a study in 2013 among a sample of smokers who have never tried e-cigarettes. The group found that ads emphasizing differences between conventional cigarettes and e-cigarettes, along with ads that showed a person using an e-cigarette, were more likely to generate interest in trying e-cigarettes among smokers than ads that did not possess those features. Future research should examine what, if any other characteristics of ads for e-cigarettes may influence use intention, actual product use, and under what circumstances these products may be used.

There are potential negative and positive public health impacts of widespread promotion and advertising of e-cigarettes. If e-cigarettes are deemed by the FDA as a potentially reduced exposure product or a smoking-cessation aid, then advertising targeted to smokers to switch to these products completely for harm reduction or cessation could be beneficial for overall public health. The data for this study showed that smokers were interested in receiving a free sample e-cigarette, regardless of advertising condition; the data also showed that 3 % of non-smokers in the e-cigarette condition displayed interest in trying e-cigarettes. Yet, when offered a free sample tobacco product, 25 % of non-smokers in the e-cigarette condition selected an e-cigarette. The current marketing environment for e-cigarettes is saturated with promotions and price discounts for these products. Such an environment could result in a greater proportion of non-tobacco users to consider trying e-cigarettes. While our data do not directly speak to increased uptake among non-tobacco users, our findings suggest low interest, and more extensive research is needed to determine the appeal of e-cigarettes to nonusers of tobacco.

### Limitations

The findings from our pilot study are subject to limitations. Primarily, this study was conducted among a fairly small sample recruited from an opt-in panel of internet users. When compared to the U.S. general population, our sample members more frequently reported having a college degree (34 % sample, 29 % population), were more likely to identify as being White, non-Hispanic (80 % sample, 64 % population), and had higher rates of cigarette (31 % sample, 18 % population) and smokeless tobacco use (8 % sample, 3 % population) [34, 36]. This affects our ability to generalize our findings to the broader population, and future research should explore this issue among a larger and more broadly generalizable sample. Yet, this sampling method also allowed us to capture more tobacco users, which is important given our findings among cigarette smokers. The sample size also limited our ability to examine these data in greater detail using multivariate modeling. An additional limitation was the use of existing advertisements for one brand of each product (Blu e-cigarettes, Camel snus). This was a result of the minimal market presence of e-cigarette advertising for other brands at the time this pilot was conducted. We cannot account for influences in intention to try that may vary based on brand loyalty, perceptions of a specific branded product, or prior exposure to the ads shown in this pilot study. As e-cigarette advertising becomes more commonplace, future studies should examine use intentions among other e-cigarette brands or other novel tobacco products. This pilot was also limited to a sample of U.S. adults aged 18 to 65. Additional research is needed to explore the impact of e-cigarette advertising and trial among a sample of youth to better inform future directions on e-cigarettes. Additionally, our study did not include baseline measures assessing attitudes about e-cigarettes or snus prior to the ads being shown to participants. Such baseline measures would have utility in better isolating effects related to product advertising or product novelty. Future studies should assess attitudes about e-cigarettes at baseline in order to better disentangle advertising effects from product attitudes. Finally, we evaluated only one form of advertising among several that exist for these types of products. However, repeated exposure to marketing through other channels and modalities may have a greater impact on perceptions, so our estimates may be lower than what may be the case for additive effects of other advertising exposures. Despite these limitations, these data are among the first we are aware of that show associations between e-cigarette advertising and interest in trial among both smokers and non-smokers.

## Conclusions

Continued promotion and advertising may peak public interest in e-cigarettes compared to snus, and may have an impact on willingness to receive promotional products or intention to try e-cigarettes. Our study found that e-cigarette advertising may influence intention to try these products, particularly among current smokers. In addition to the absence of an established safety profile and regulation on the quality of e-cigarettes, additional considerations about product advertising should be considered in developing future regulatory actions.

## Additional file

Additional file 1.
**Advertisements shown in exposure groupings.** Blu e-cigarette and Camel snus ads shown in exposure conditions.

## Abbreviation

SLT: Smokeless tobacco
