# Supplementary material for: Associations between perceptions of e-cigarette advertising and interest in product trial amongst US adult smokers and non-smokers: results from an internet-based pilot survey
Source: Tob Induc Dis. 2015 Jun 12;13(1):14. doi: 10.1186/s12971-015-0039-6 (PMC4502389; doi:10.1186/s12971-015-0039-6)

## Slide 1
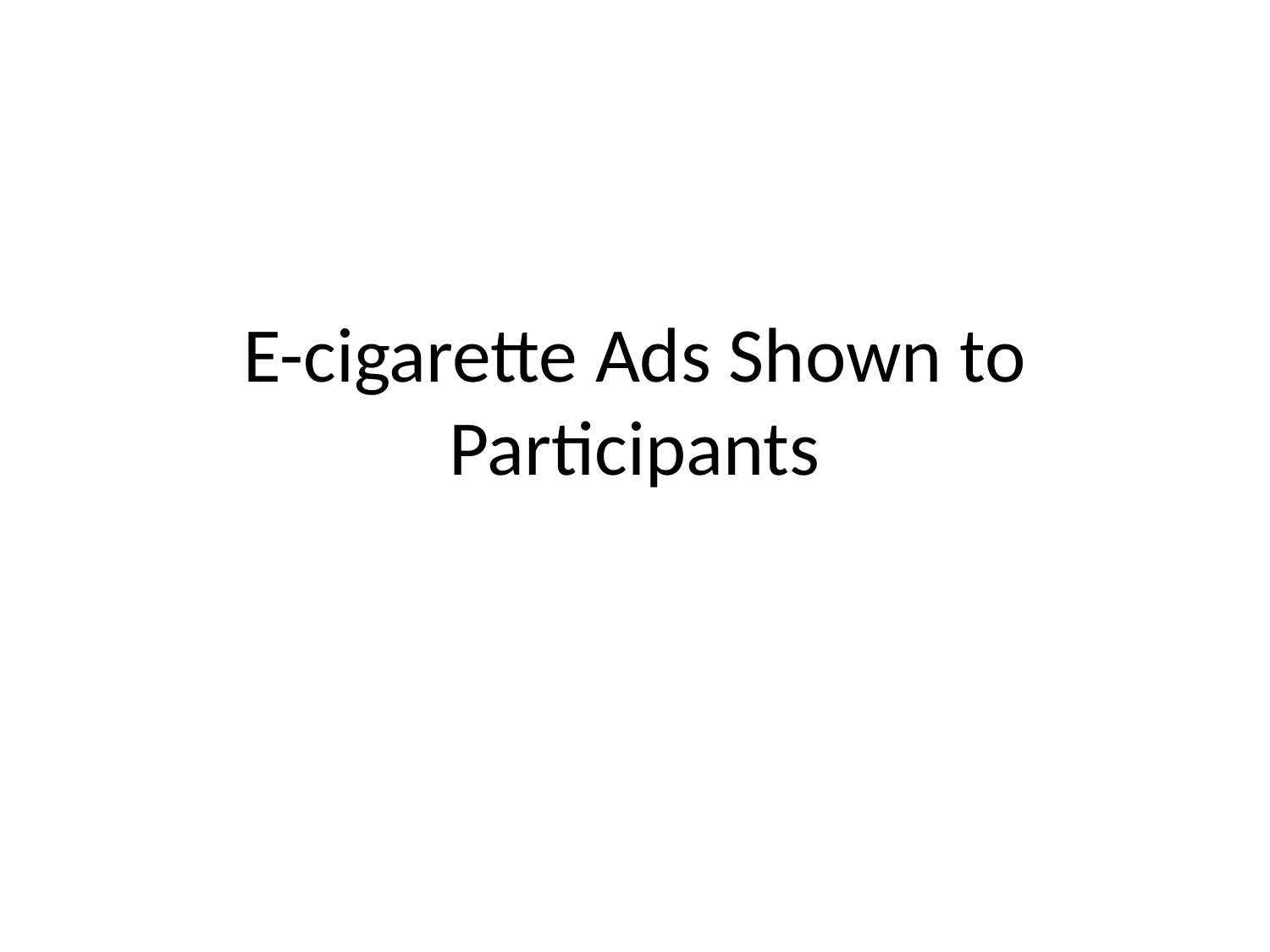

# E-cigarette Ads Shown to Participants

## Slide 2
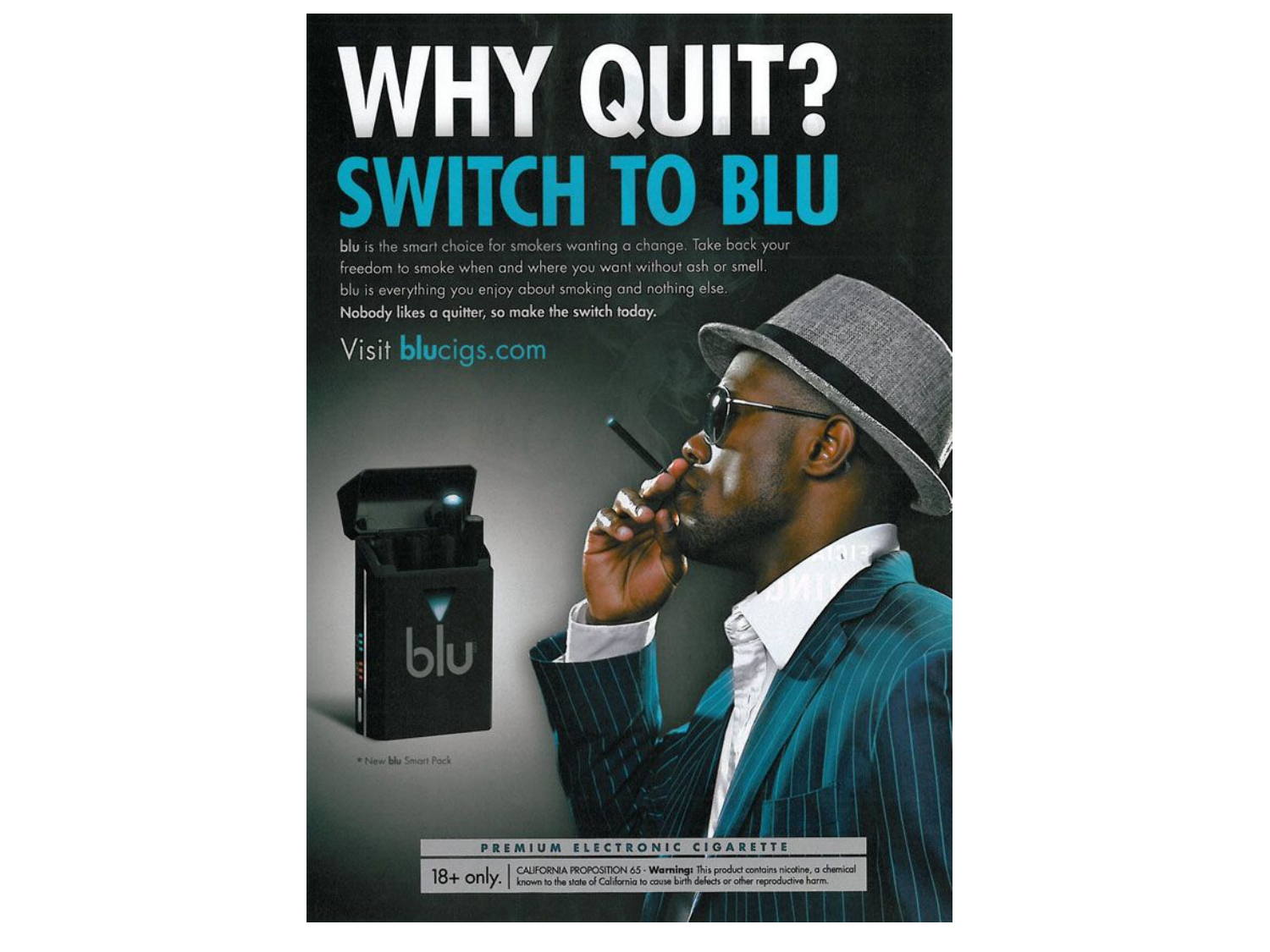

## Slide 3
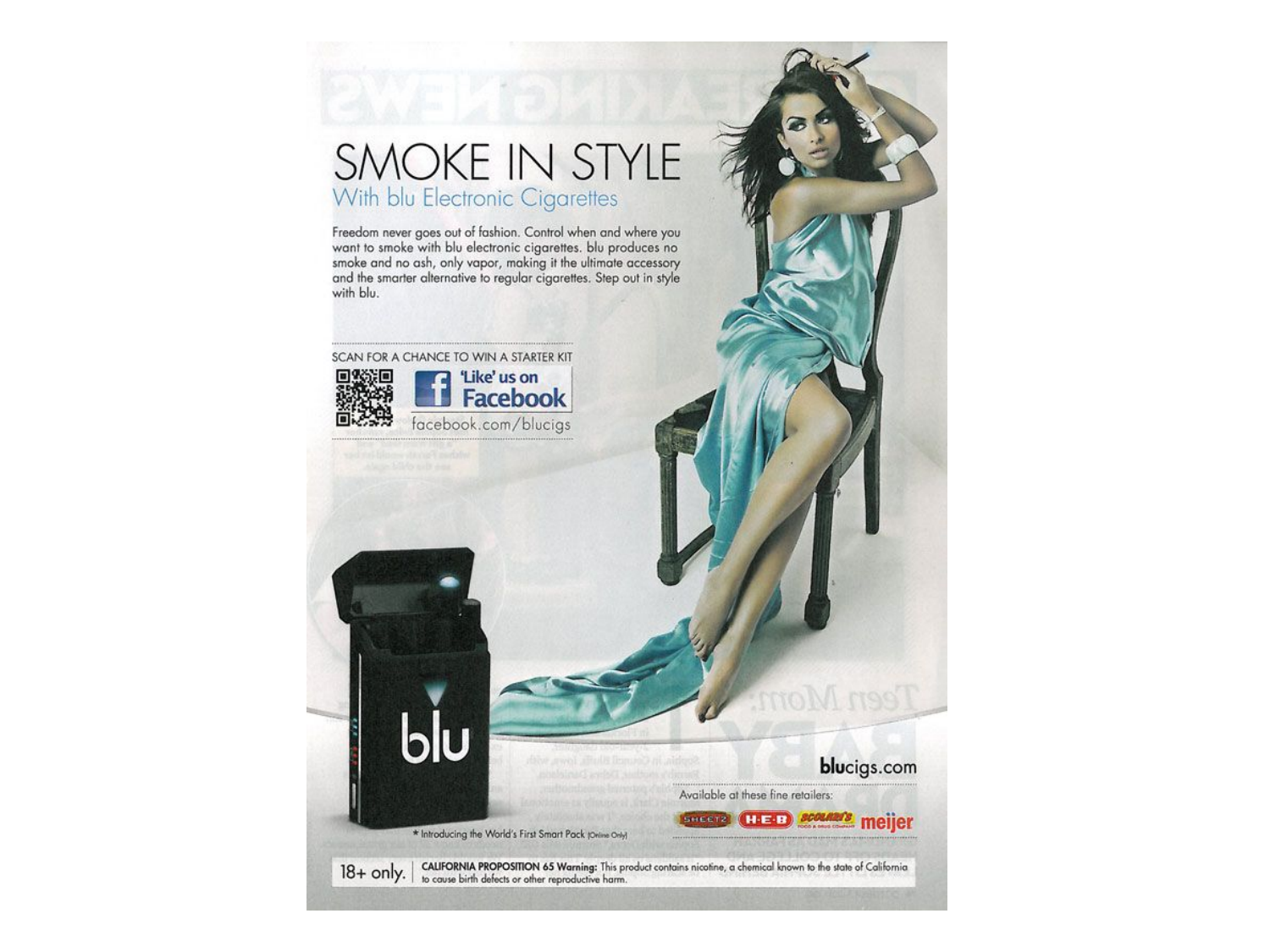

## Slide 4
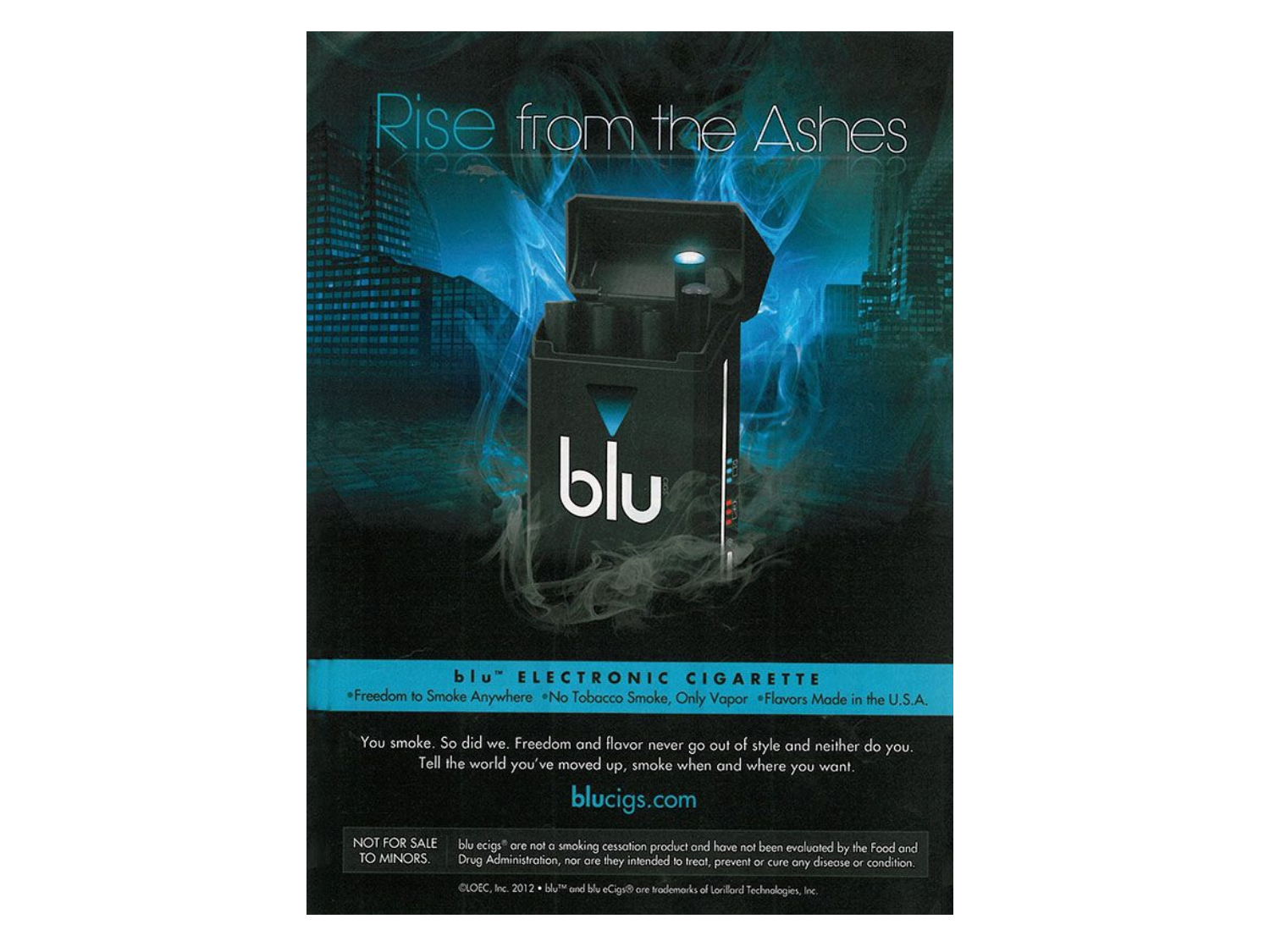

## Slide 5
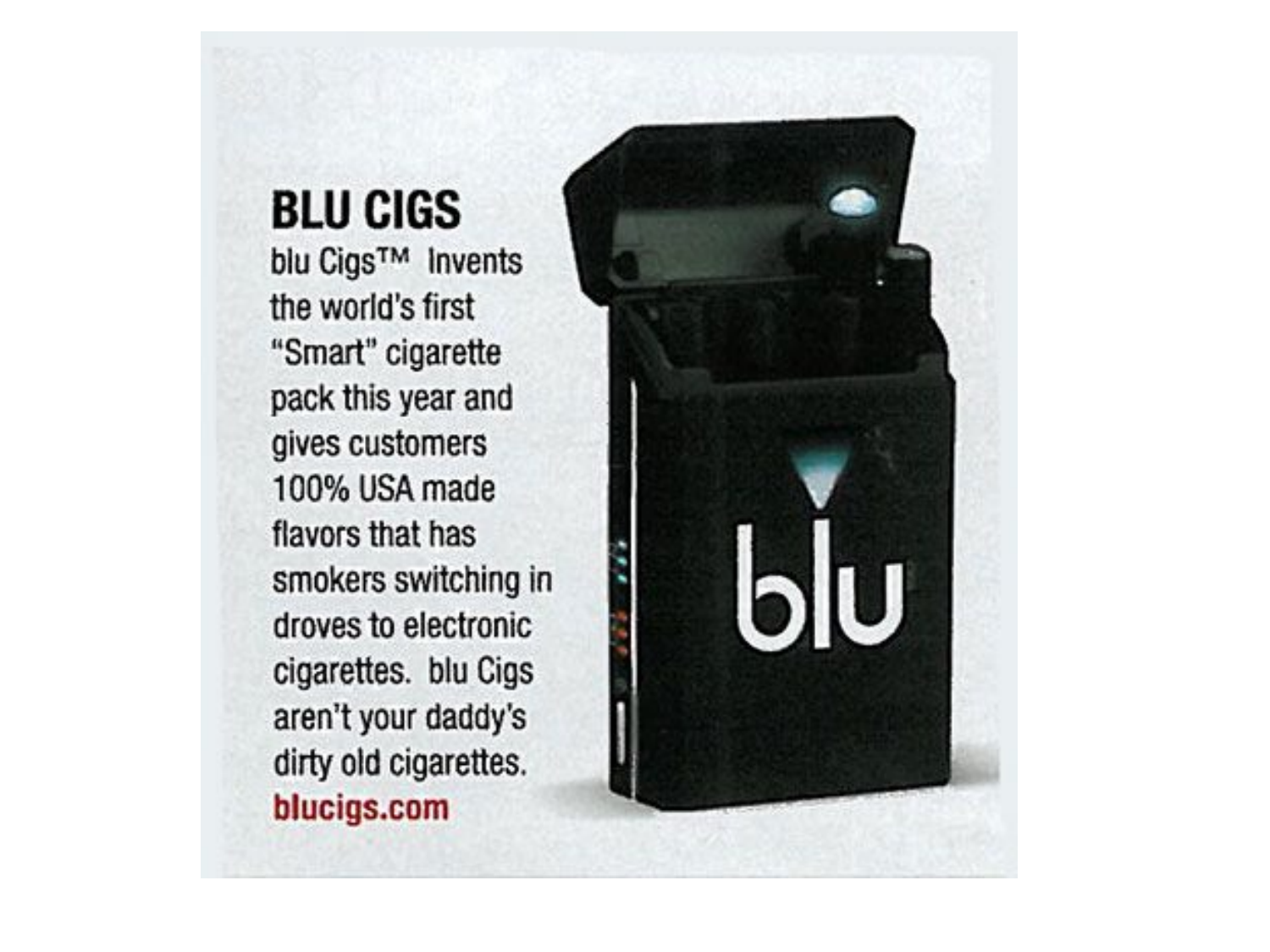

## Slide 6
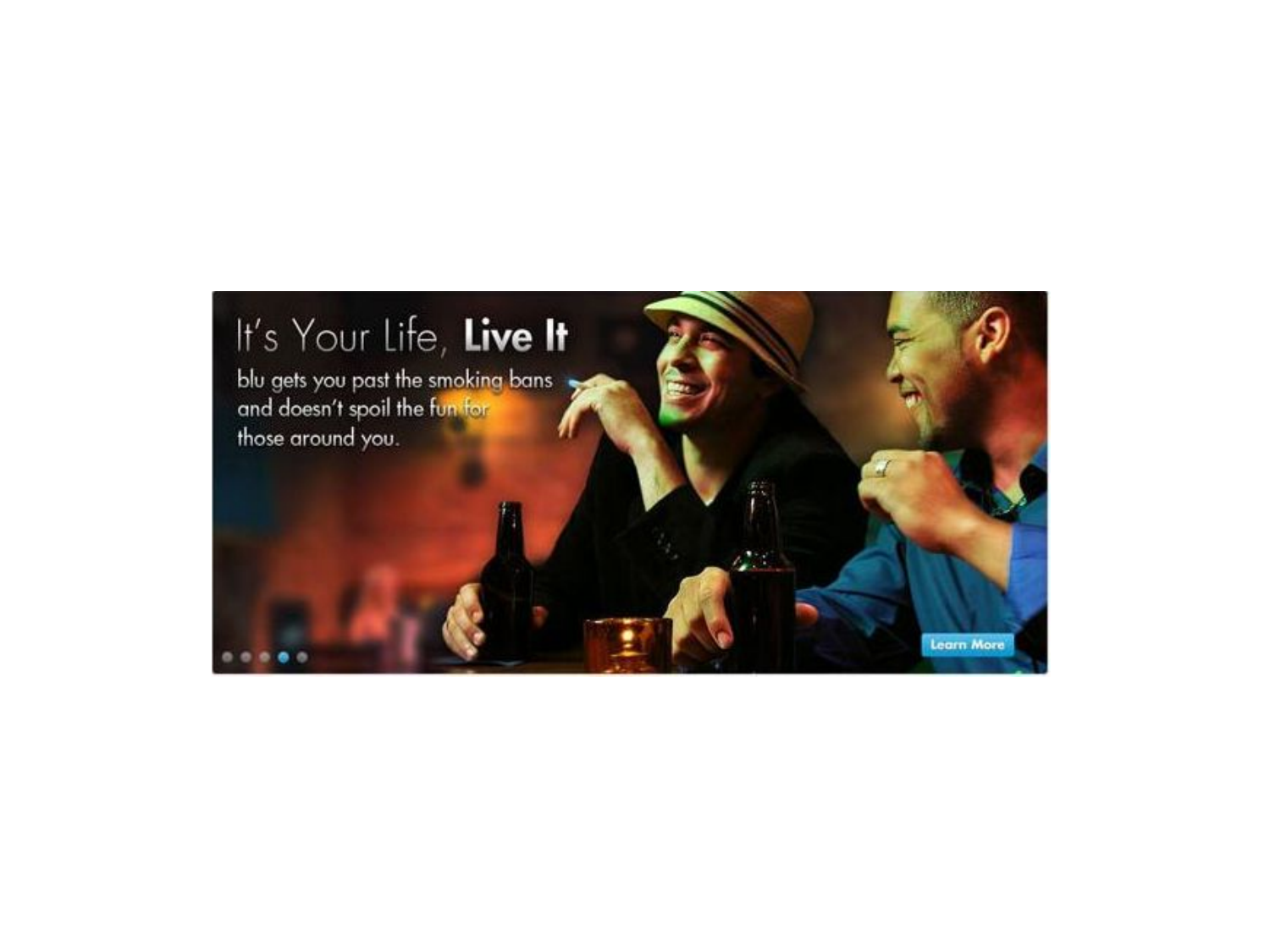

## Slide 7
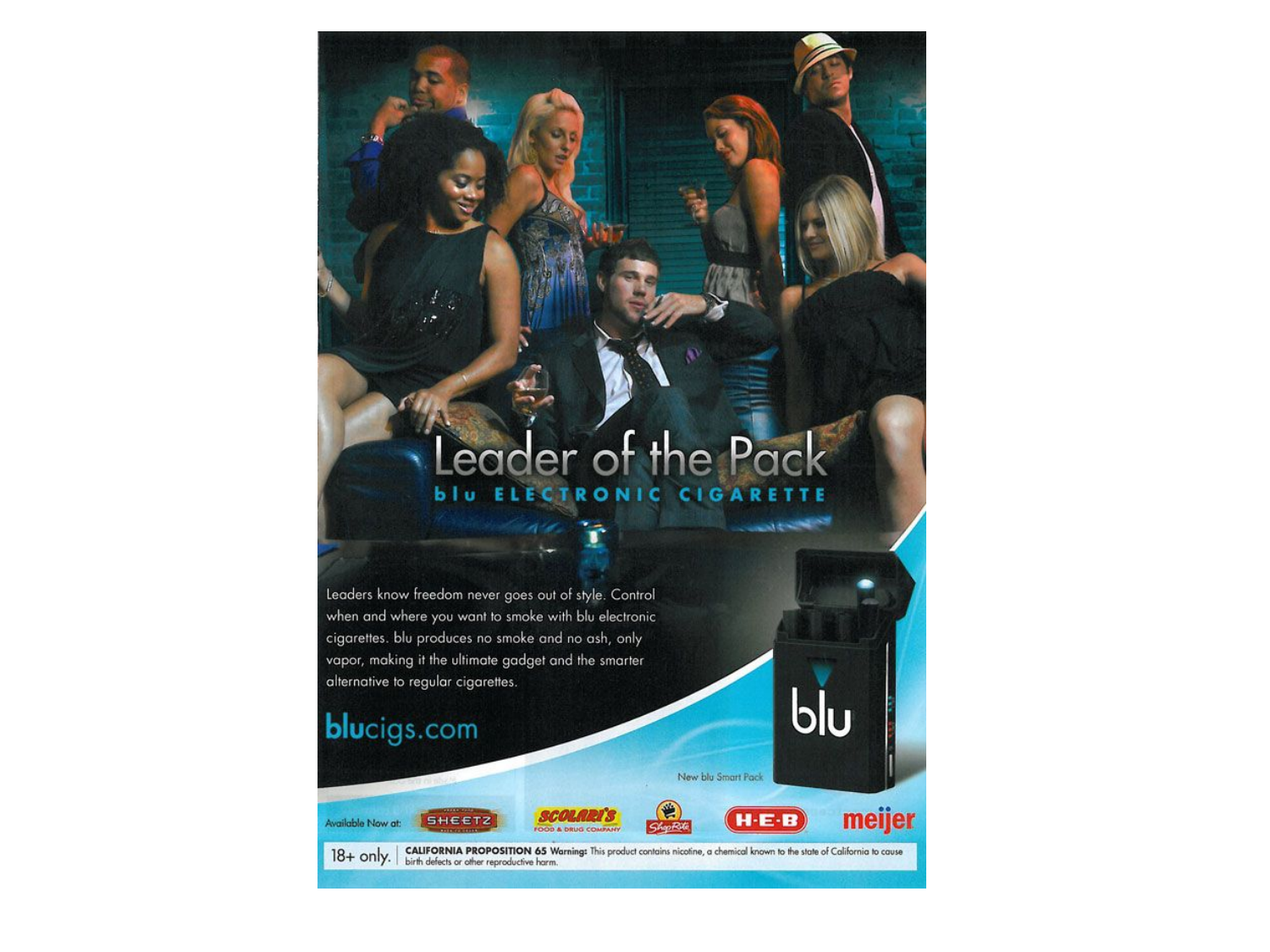

## Slide 8
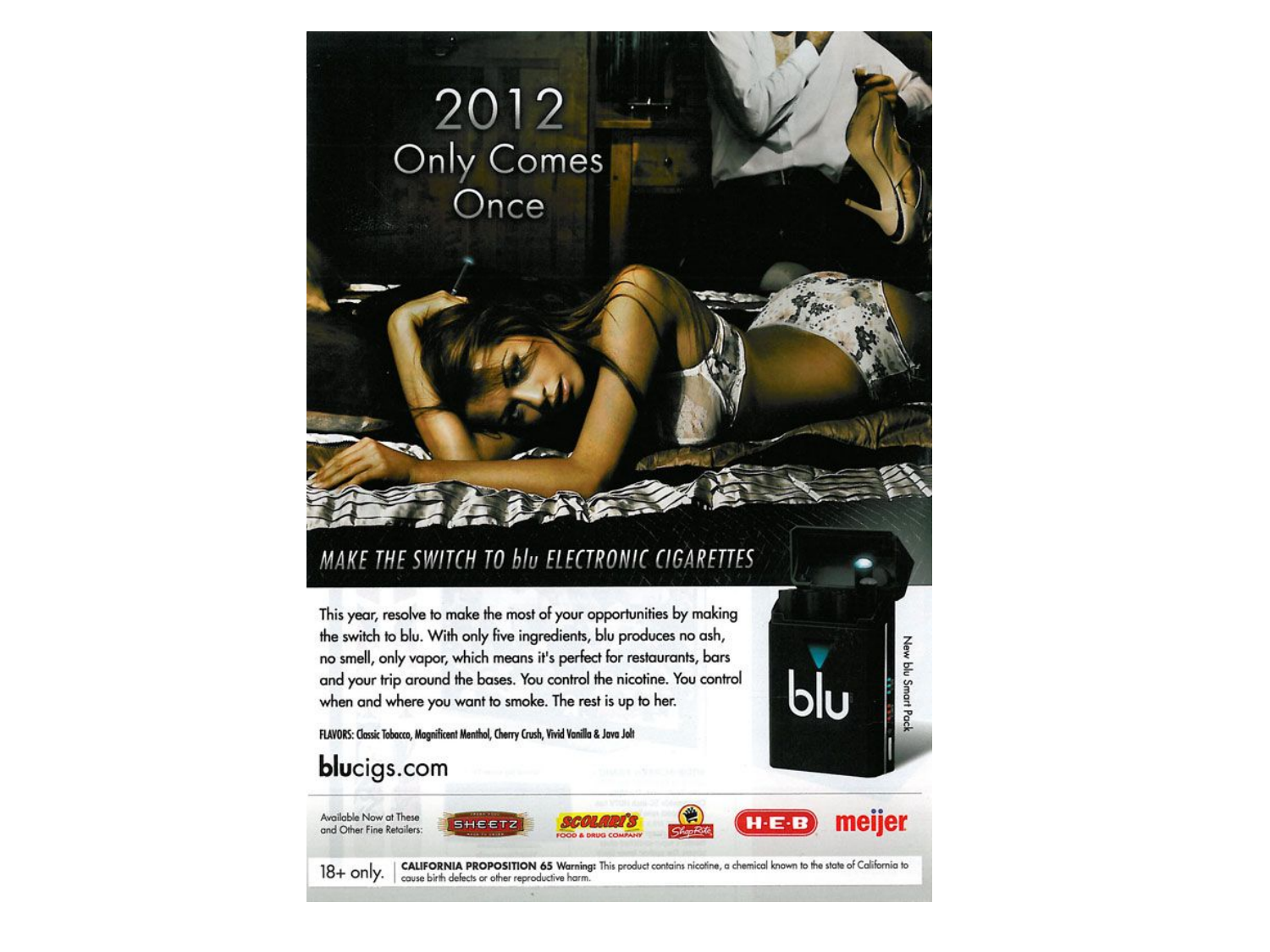

## Slide 9
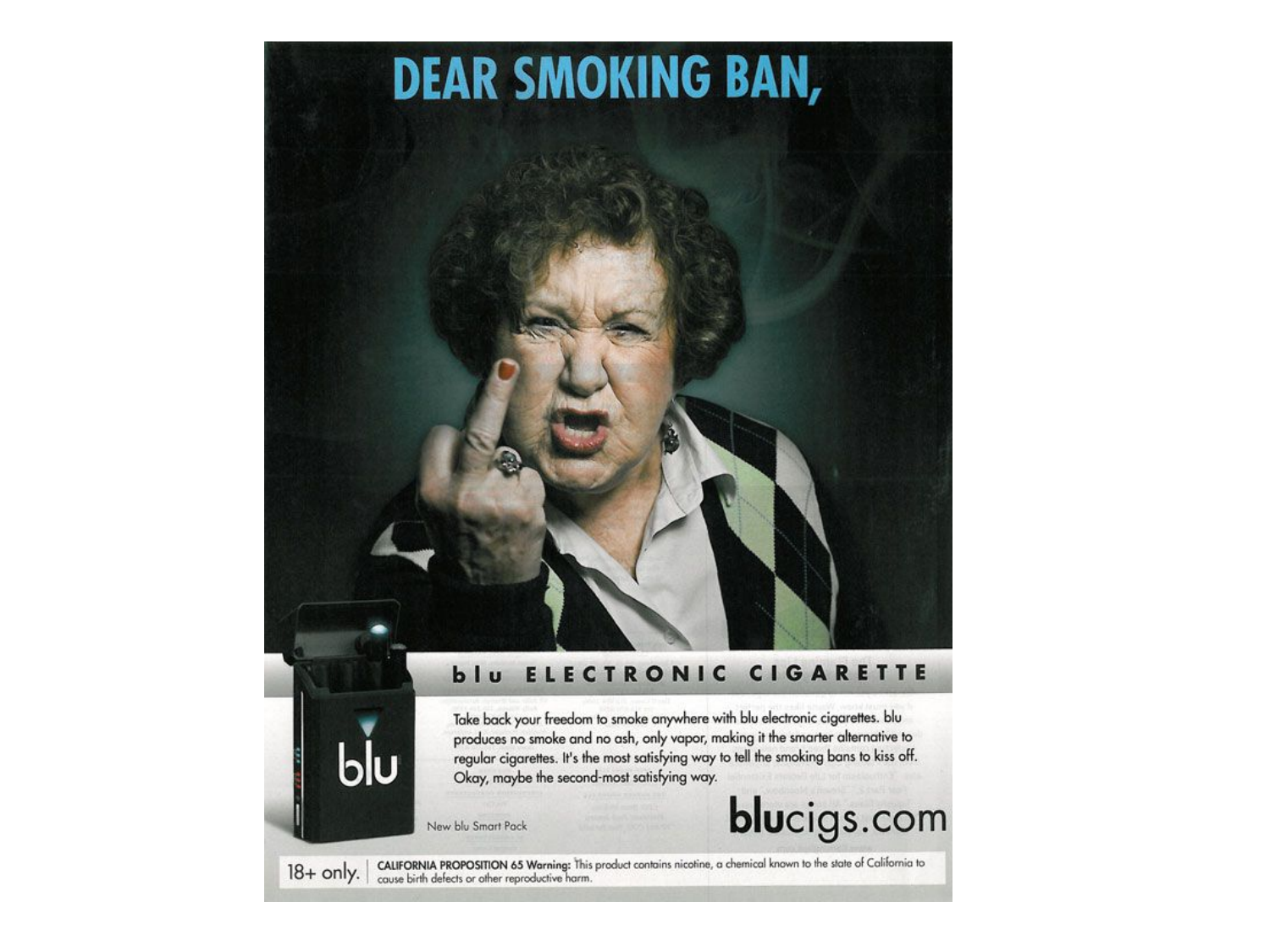

## Slide 10
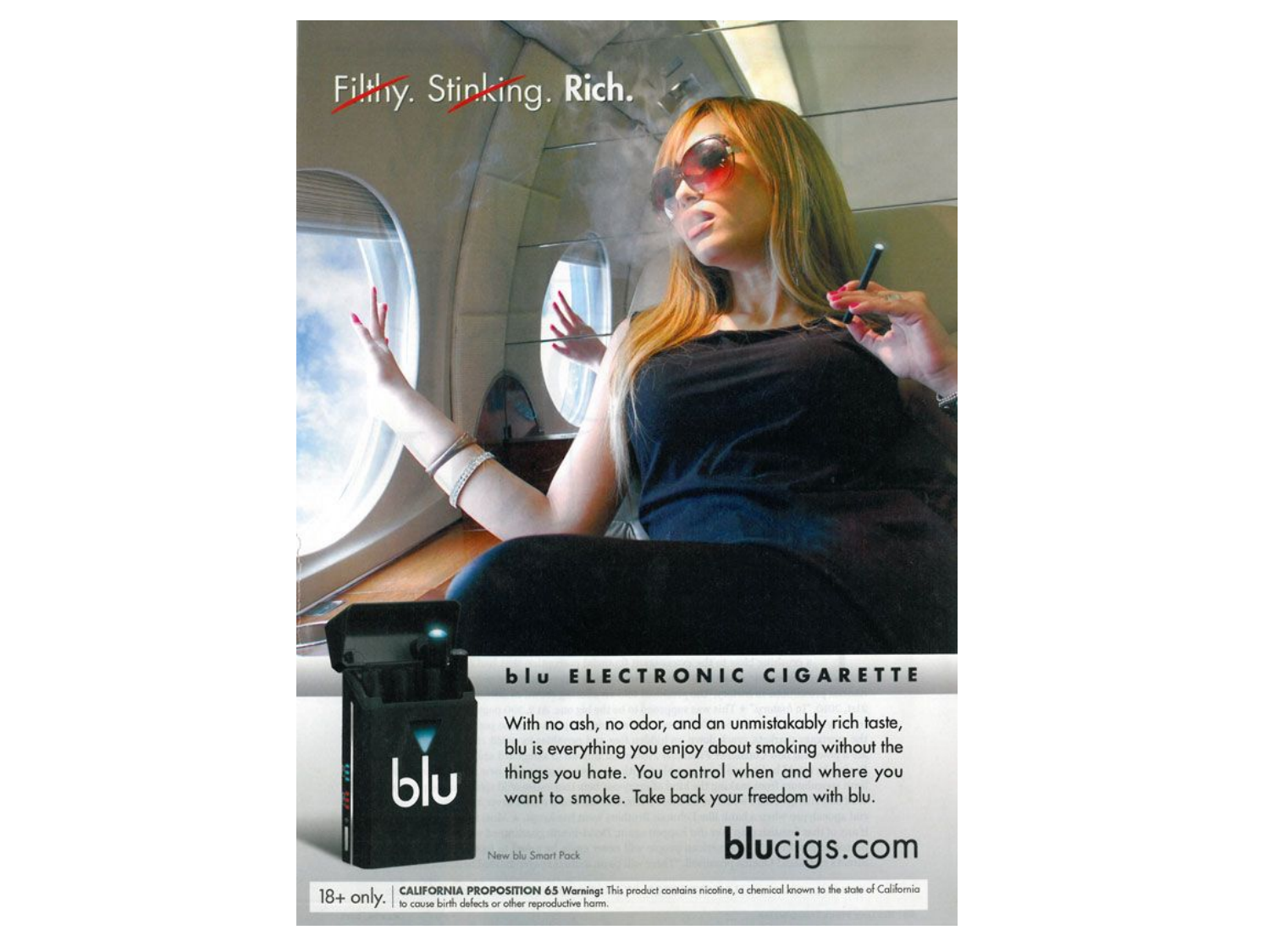

## Slide 11
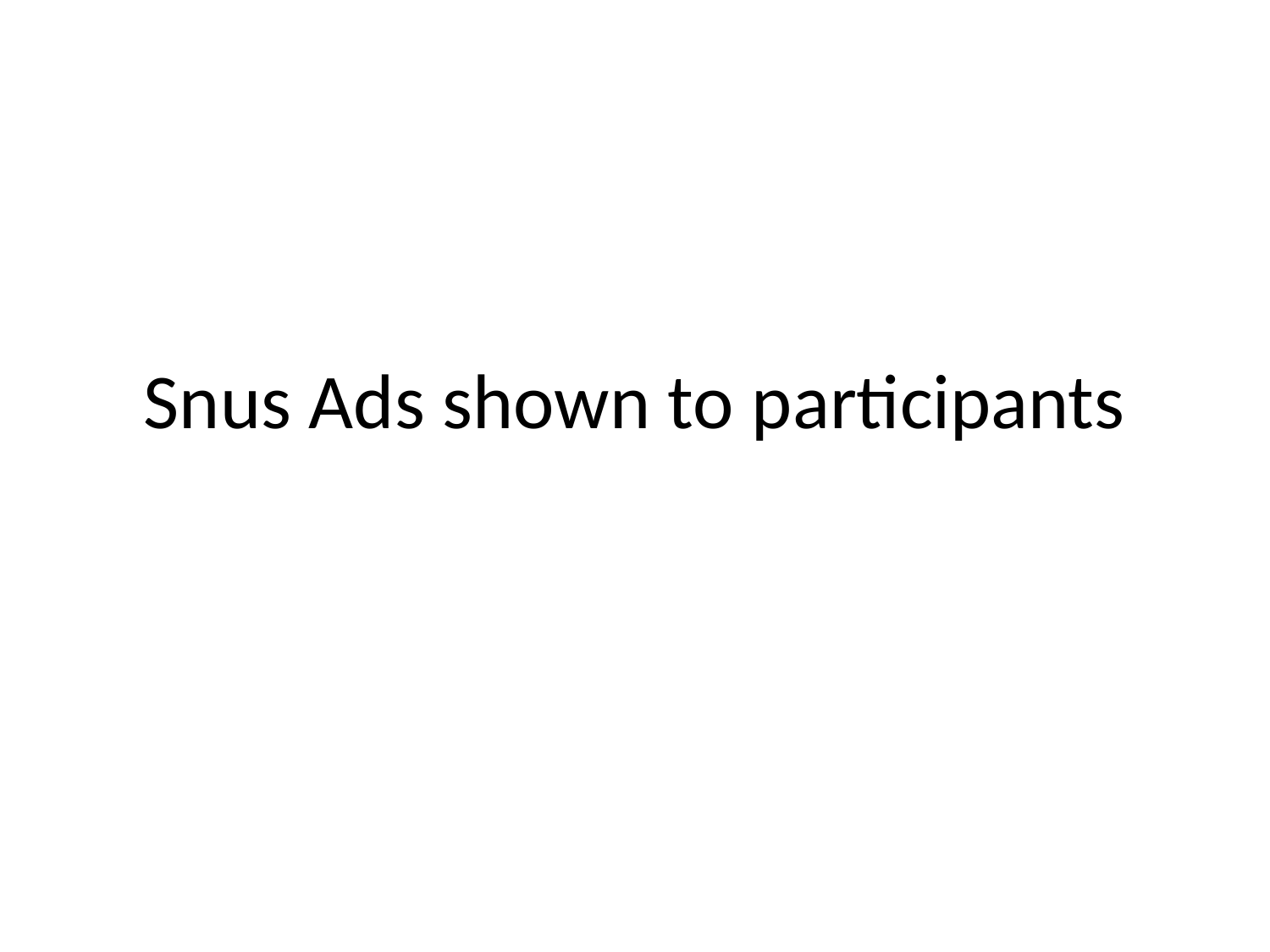

# Snus Ads shown to participants

## Slide 12
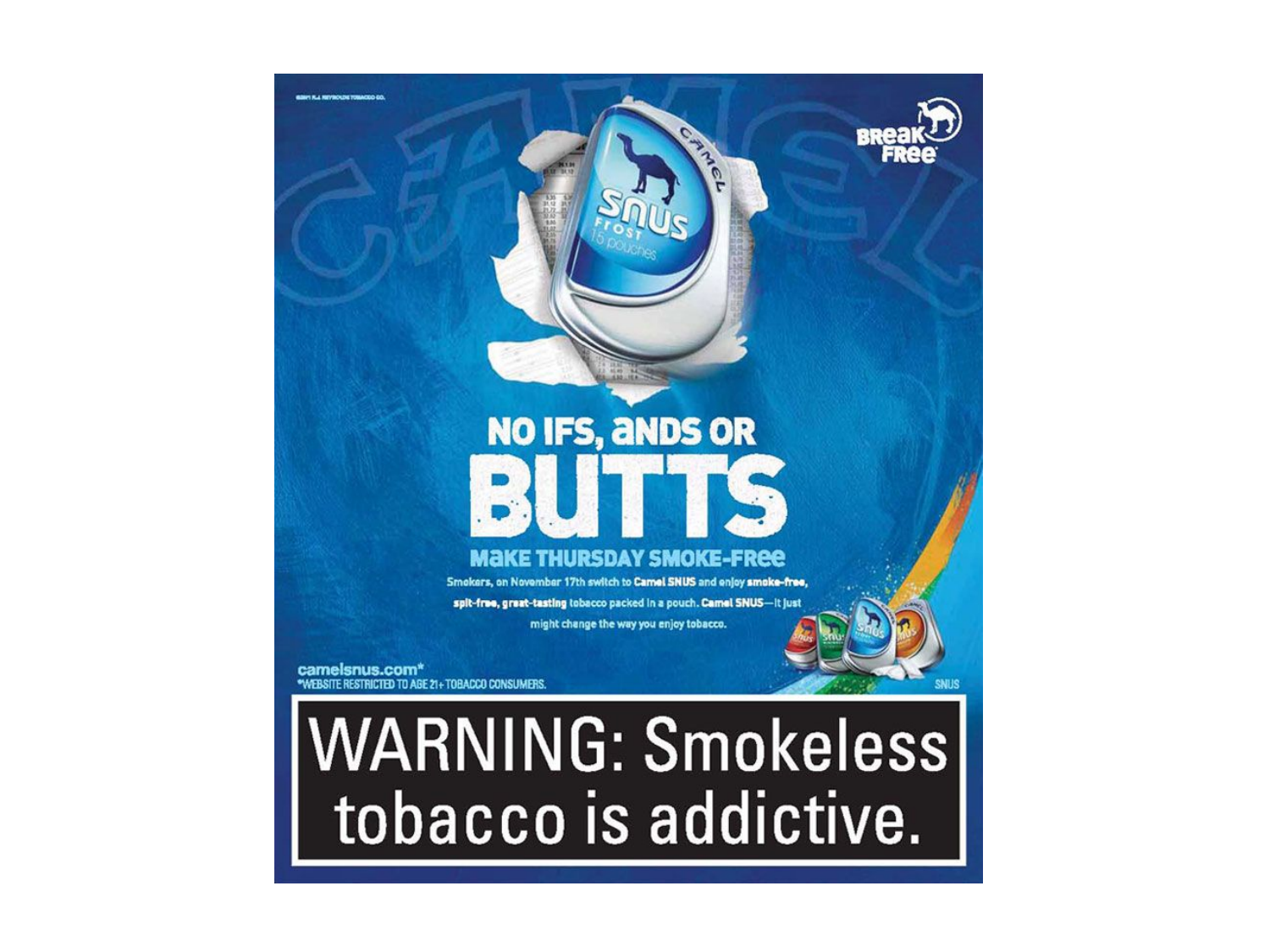

## Slide 13
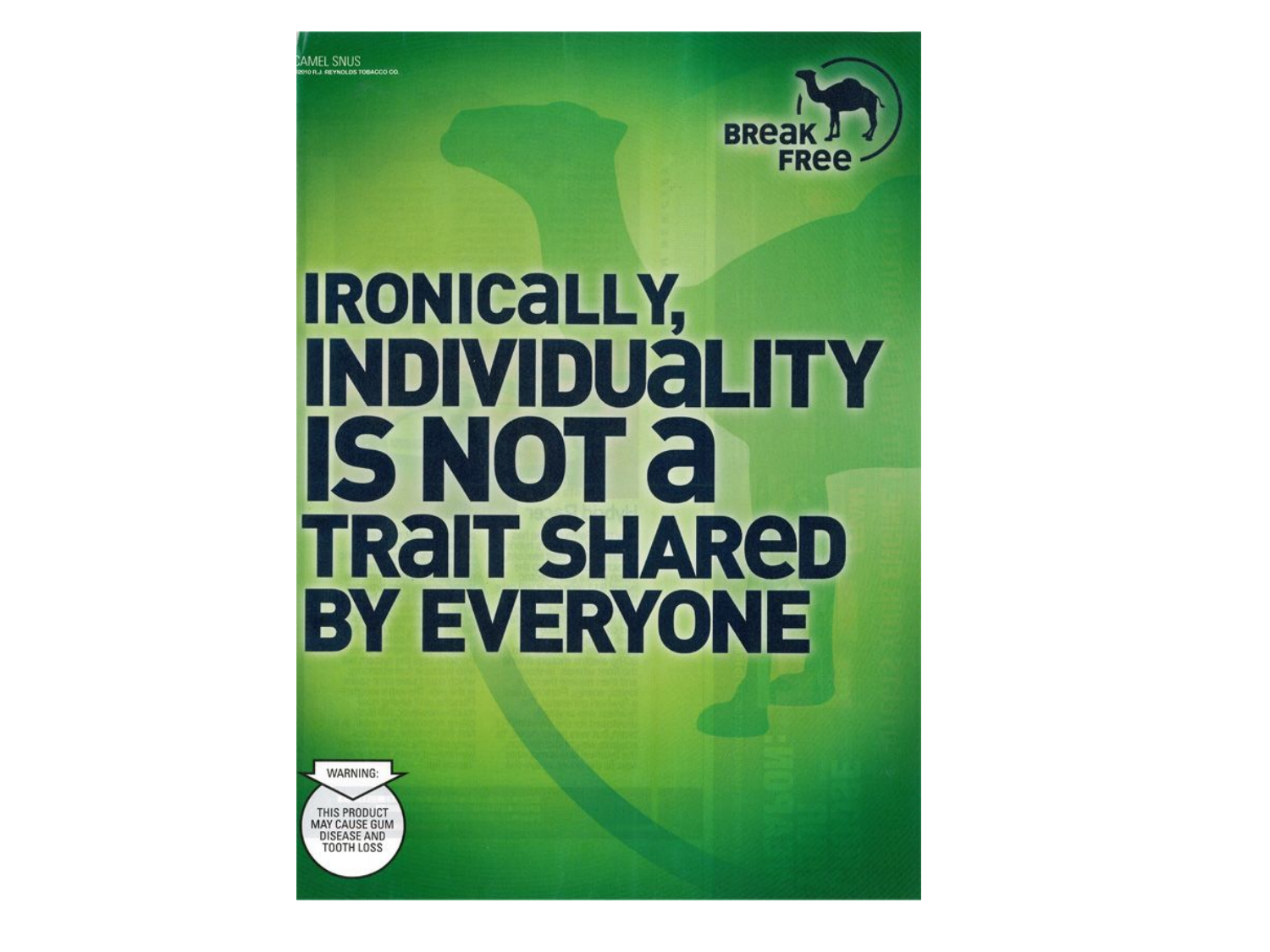

## Slide 14
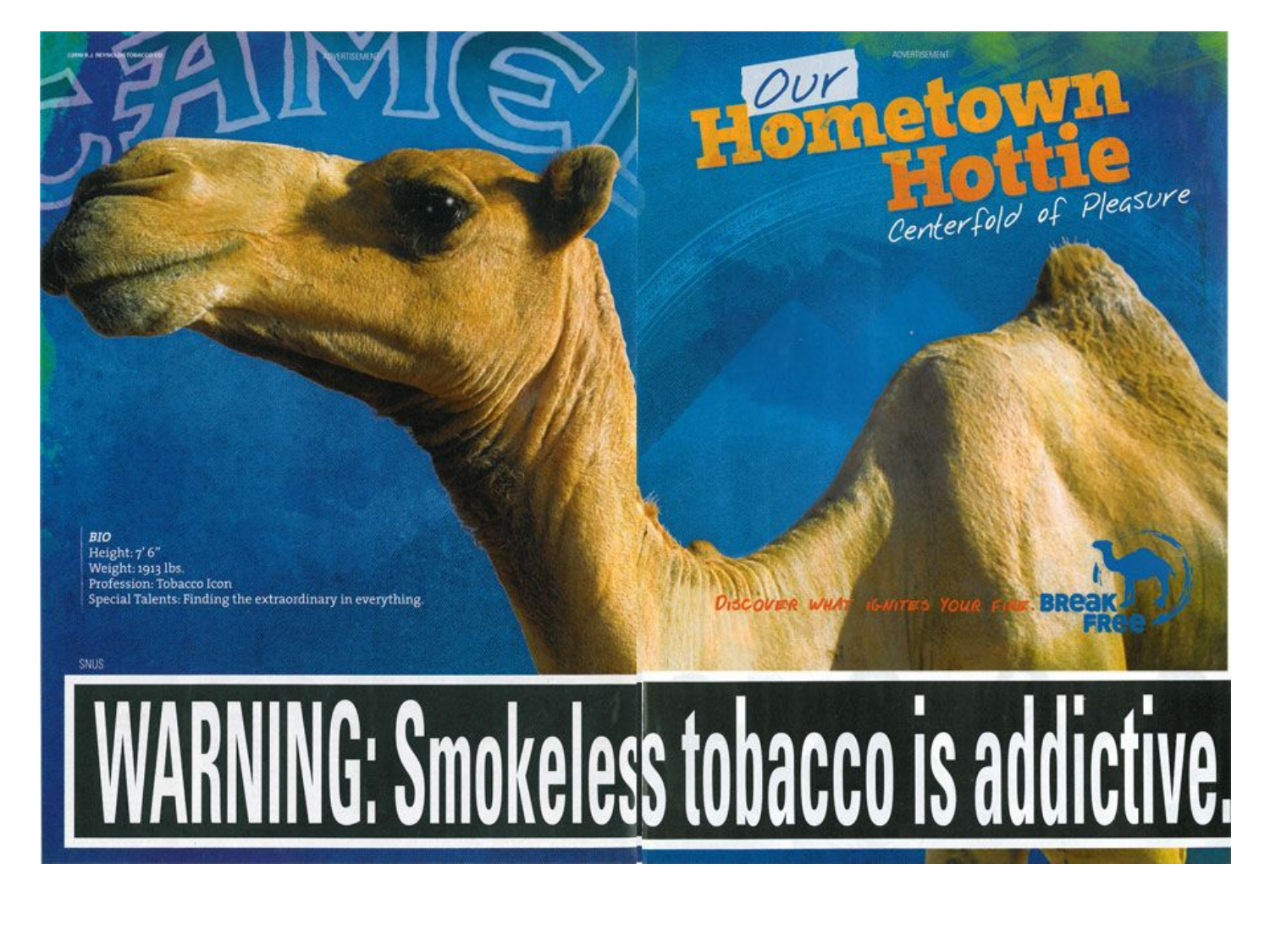

## Slide 15
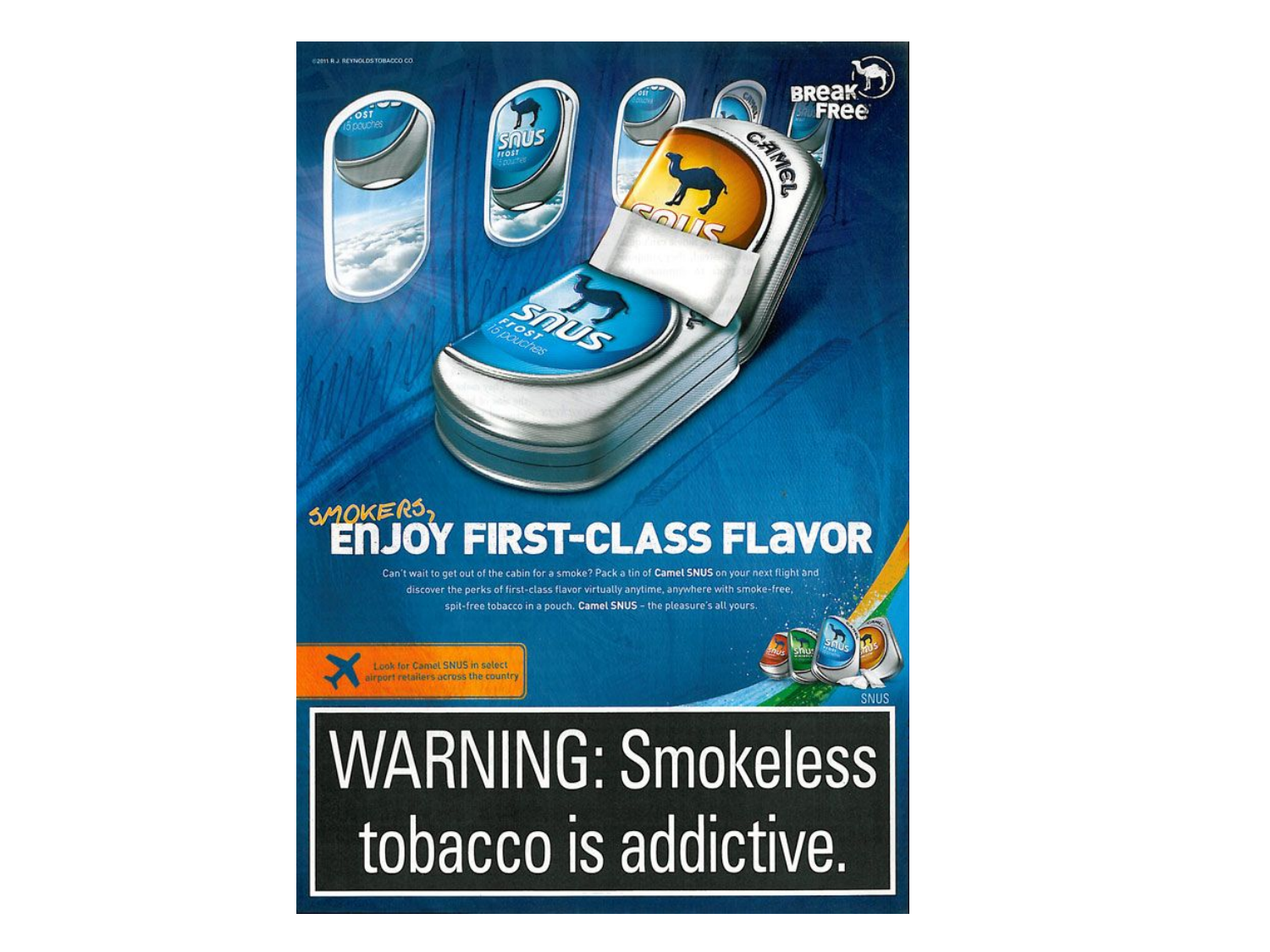

## Slide 16
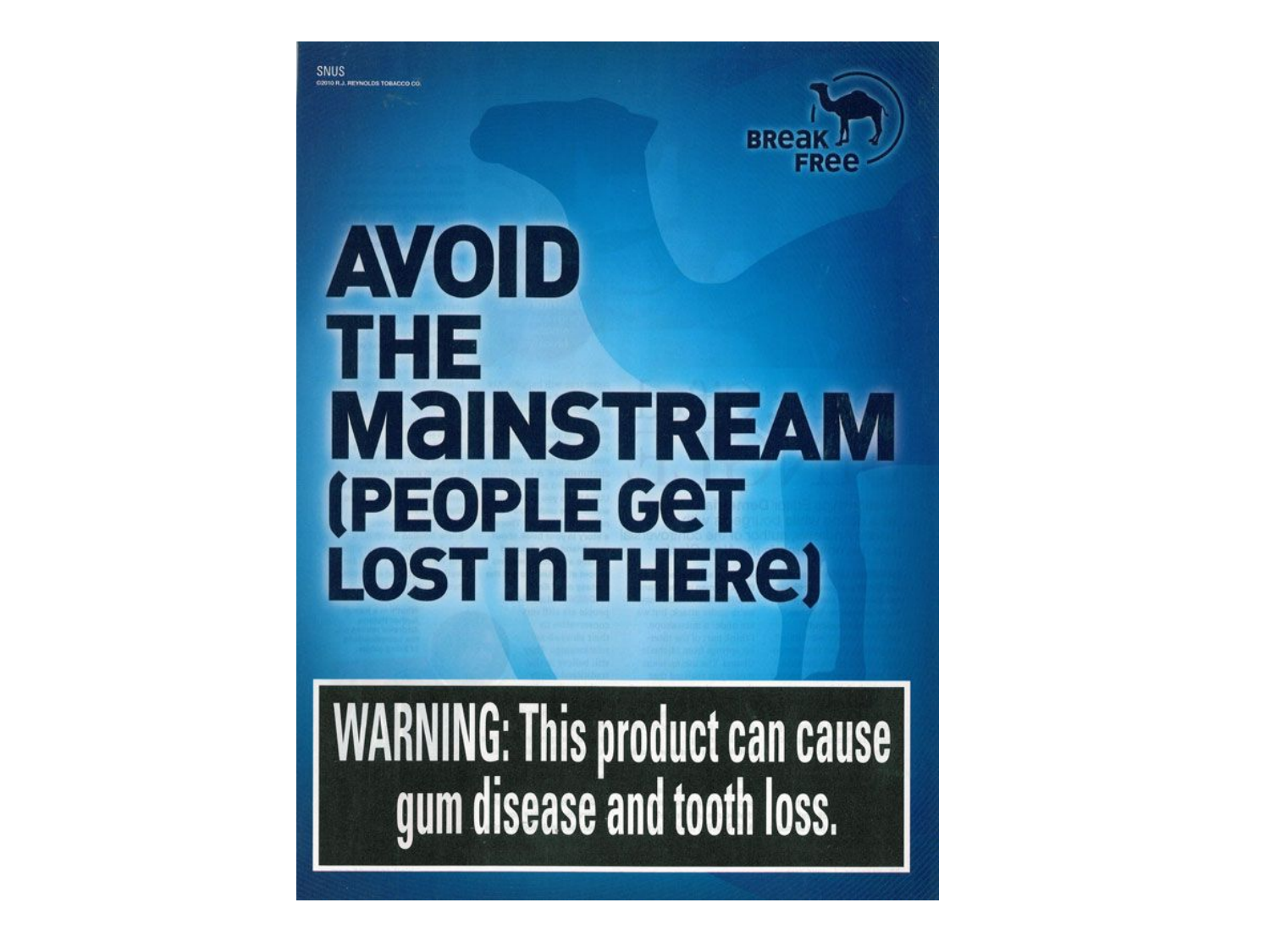

## Slide 17
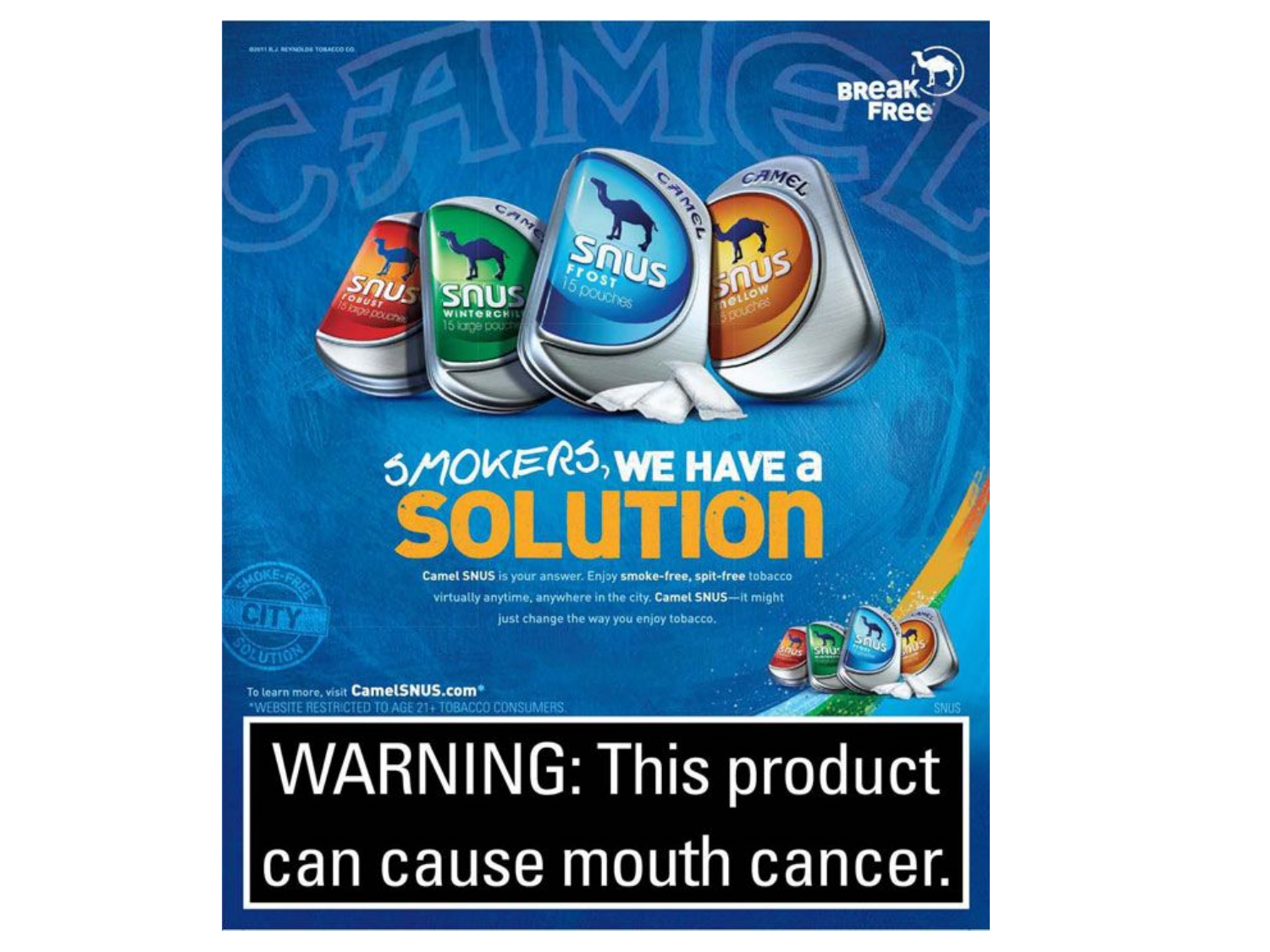

## Slide 18
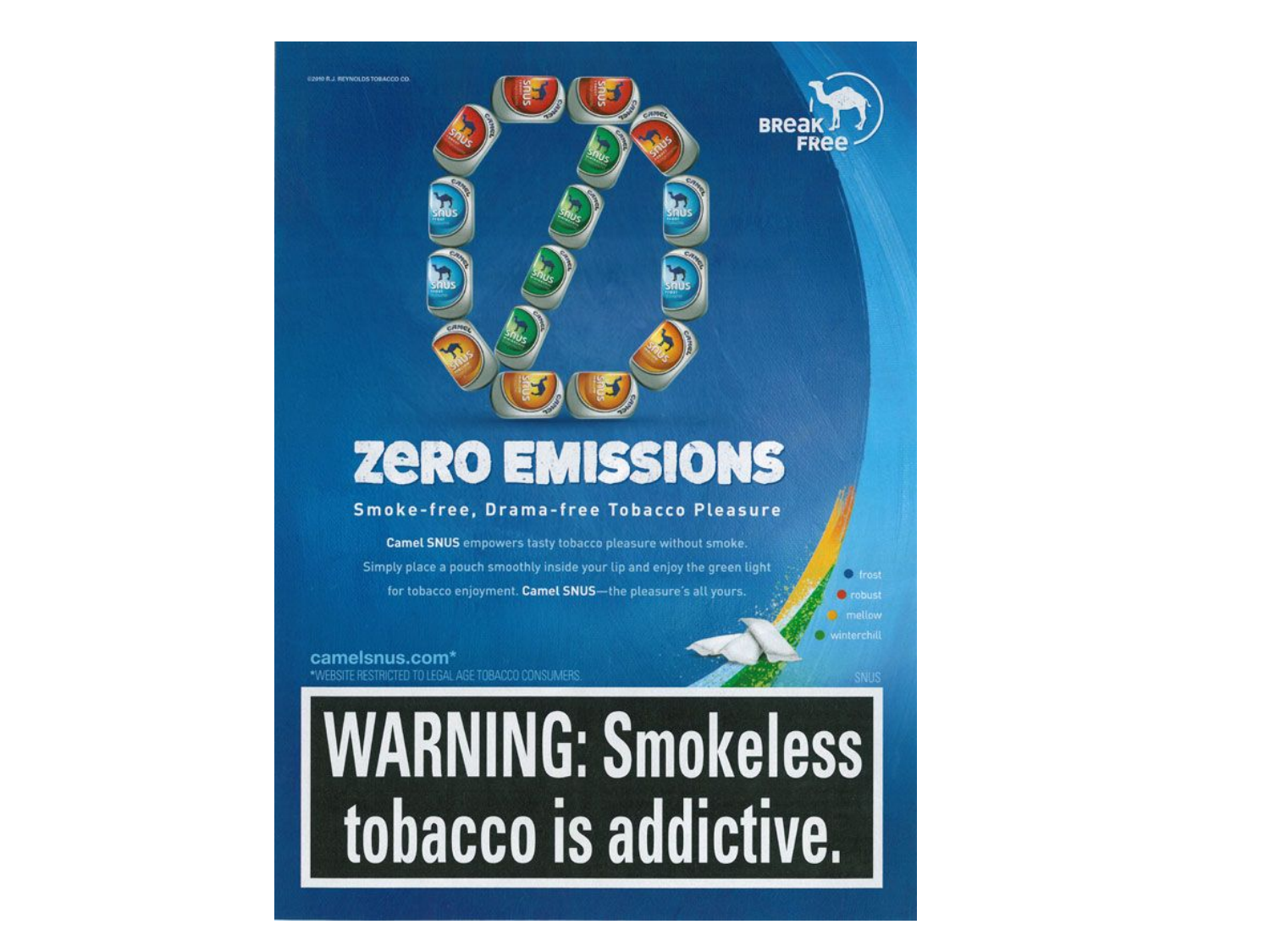

## Slide 19
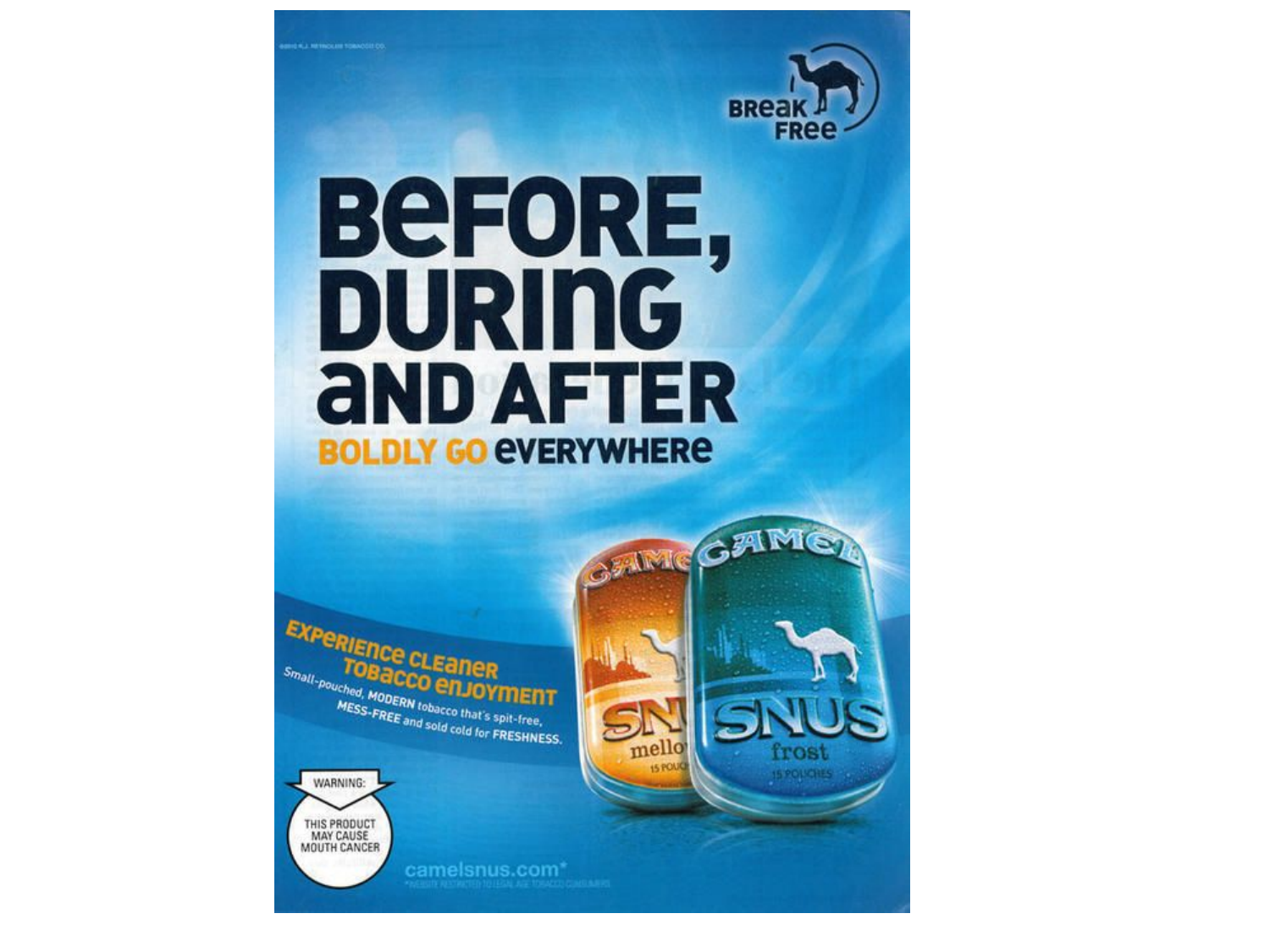

## Slide 20
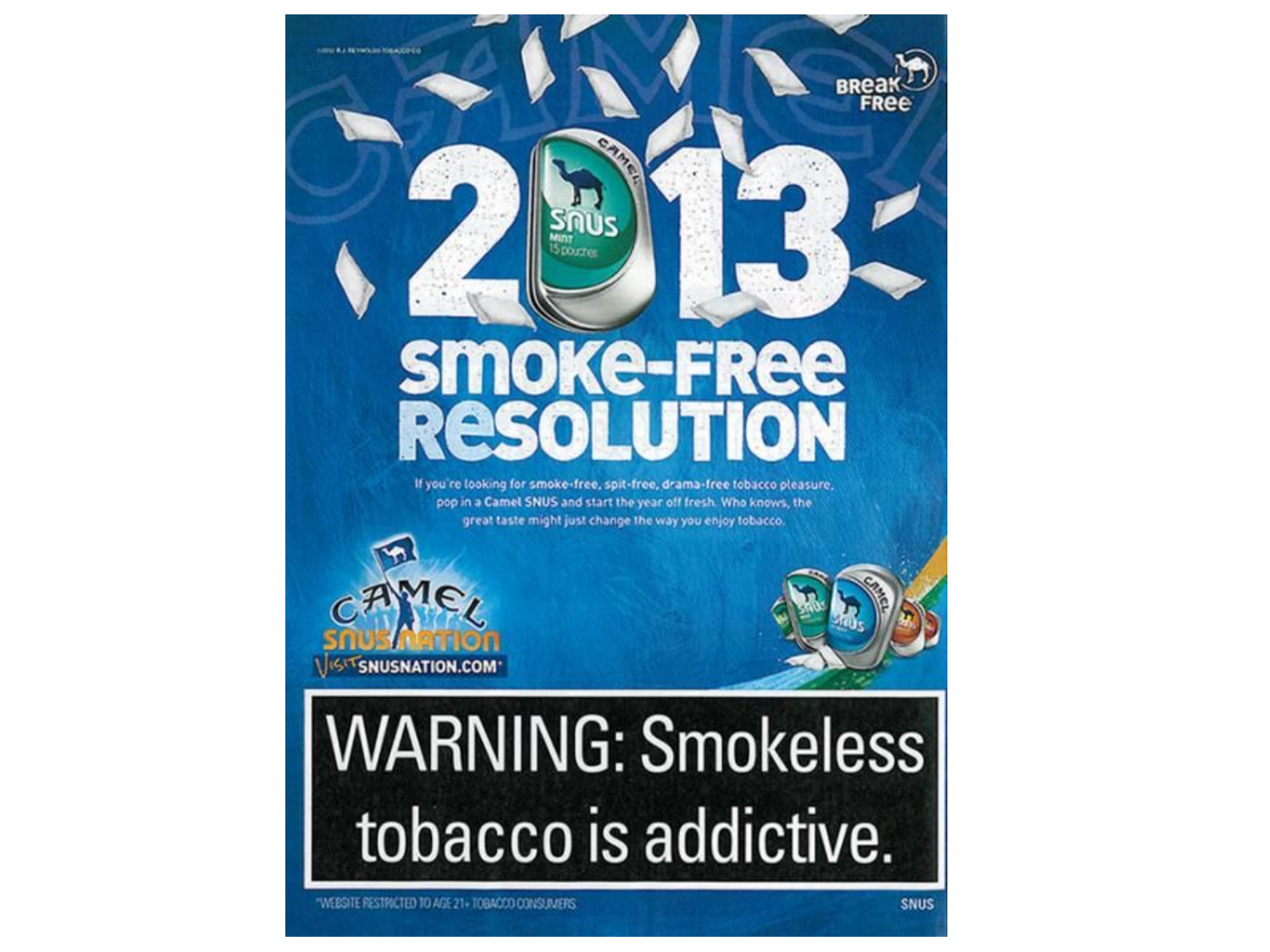

Supplement: Additional file 1. — Advertisements shown in exposure groupings. Blu e-cigarette and Camel snus ads shown in exposure conditions. [file 12971_2015_39_MOESM1_ESM.pptx]
